# Polysaccharide-degrading archaea dominate acidic hot springs: genomic and cultivation insights into a novel *Thermoproteota* lineage

**DOI:** 10.1128/msystems.00710-25

**Published:** 2025-09-22

**Authors:** Maria I. Prokofeva, Alina I. Karaseva, Adolf S. Tulenkov, Alexandra A. Klyukina, Natalia E. Suzina, Nicole J. Bale, Anchelique Mets, Christa Schleper, Alexander G. Elcheninov, Tatiana V. Kochetkova

**Affiliations:** 1 Federal Research Centre “Fundamentals of Biotechnology” of the Russian Academy of Sciences, Moscow, Russia; 2 Moscow Center for Advanced Studies, Moscow, Russia; 3 G.K. Skryabin Institute of Biochemistry and Physiology of Microorganisms of the Russian Academy of Sciences, Federal Research Center “Pushchino Scientific Center for Biological Research of the Russian Academy of Sciences”, Pushchino, Moscow region, Russia; 4 Department of Marine Microbiology and Biogeochemistry, NIOZ Royal Netherlands Institute for Sea Research, Den Burg, the Netherlands; 5 Department of Functional and Evolutionary Ecology, Archaea Biology and Ecogenomics Unit, University of Vienna, Vienna, Austria; Monash University, Melbourne, Victoria, Australia

**Keywords:** *Marsarchaeota*, *Tardisphaera*, archaea, thermoacidophile, polysaccharides metabolism, glycosidases

## Abstract

The expansion of sequencing technologies and bioinformatics has greatly advanced our understanding of microbial “dark matter,” yet the isolation of pure cultures, especially among Archaea, remains rare and challenging. Cultivation is still essential for the reliable characterization of microbial metabolism, which cannot be fully replaced by metagenomics and other omics-based approaches. Here, we report the first cultivated representatives of a deep-branching archaeal lineage previously known as *Candidatus* Marsarchaeota. Our phylogenomic analyses place these isolates within the phylum *Thermoproteota* as a novel order, *Tardisphaerales*. Members of *Tardisphaerales* dominate the prokaryotic communities in acidic hot springs below 70°C, comprising up to 40% of the total microbial population, underscoring their ecological significance. Functional genomics and culture experiments reveal a thermoacidophilic, anaerobic lifestyle, with energy metabolism based on carbohydrate fermentation, particularly of polysaccharides. This metabolic capability is supported by numerous glycosidase-encoding genes and by unprecedented metabolic versatility among thermoacidophiles. The isolates possess complete glycolysis, Entner-Doudoroff, and pentose-phosphate pathways, allowing them to utilize different sugars. Specialization in polysaccharide hydrolysis presumably provides an adaptive advantage for these slow-growing archaea, as most other heterotrophic thermoacidophiles prefer peptides or simple sugars. Furthermore, robust defense mechanisms against reactive oxygen species and persistence in acidic conditions enable *Tardisphaerales* to outcompete other heterotrophs and maintain dominance in these extreme habitats. The discovery and cultivation of this new order expand prokaryotic taxonomy and reveal the key players in carbon cycling in acidic geothermal ecosystems.

**IMPORTANCE:**

Most of the dominant prokaryotes in natural environments remain uncultivated, and their metabolic potential and ecological role can be inferred solely from metagenomics. However, cultivation is essential for comprehensive functional characterization and identification of novel traits. Here, we describe the first cultivated representatives of the new archaeal order *Tardisphaerales* within the novel class *Tardisphaeria* (phylum *Thermoproteota*), a lineage abundant in acidic hot springs. Through the whole-genome reconstruction and microbiological experiments in pure cultures, we demonstrate that these archaea are metabolically distinct from the known thermoacidophiles, making them the key degraders of the complex organic matter in hot, acidic environments. Their genomes encode a diverse set of glycosidases that allow efficient polysaccharide breakdown at high temperatures and low pH, a trait with promising biotechnological applications.

## INTRODUCTION

Acidic hot springs, surrounding soils, and solfataras closely associated with volcanic outflows or calderas are inhabited by specific thermoacidophilic prokaryotes adapted to live at low pH values (<5 pH_opt_) and high (≥45°C) temperatures (1). The microbial community of such environments consists of chemolithoautotrophic species living in hot water and heterotrophic ones settling mostly in sediments (2). The latter are dominated by archaea, both representatives of the cultivated taxa, such as *Thermoplasma*, *Sulfolobus, Saccharolobus*, *Caldisphaera* (3), *Acidilobus* (4), and *Conexivisphaera* (5), as well as still unexplored deep lineages (2, 6–9).

Thermoacidophilic archaea can dominate in such extreme conditions due to an arsenal of adaptations: maintaining pH homeostasis by a reverse membrane potential formed by the influx of K^+^, the action of primary and secondary transporters, possessing a highly impermeable tetraether-based membrane (10), defenses against heavy metals and reactive oxygen species (ROS) extensively produced under acidic conditions (11, 12). Importantly, all isolated thermoacidophilic prokaryotes use the efficient energy-producing machinery, based on the tricarboxylic acid (TCA) cycle and the respiratory chains, since life under polyextreme conditions is energetically expensive (13).

Most of the known heterotrophic thermoacidophiles metabolize proteinaceous substrates and/or simple sugars. The investigated thermoacidophilic archaea harbor modified variants of the Embden-Meyerhoff-Parnas (EMP) and/or the Entner-Doudoroff (ED) pathways (14) to catabolize sugars. Pentose degradation in archaeal cells differs significantly from that in bacteria. In *Sulfolobus* and *Saccharolobus* (and some haloarchaea), the degradation of C_5_-sugars proceeds via conversion to 2-oxoglutarate, and it has been shown that only *Halorhabdus* species utilize pentose via the non-oxidative pentose-phosphate pathway (PPP) (15).

In geothermal habitats, organic matter decomposition primarily initiates with degradation of polysaccharides (PSs), which originate from prokaryotic extracellular polymeric substances, cell wall components, and external organic matter such as remnants of higher plants, fungi, lichens, and other matter transported from surrounding areas (16–18). However, data on the growth of thermoacidophilic prokaryotes on PSs are limited. To date, only some acidophilic archaea from hyperthermophilic genera, like *Acidilobus*, *Caldisphaera,* and *Saccharolobus,* are known to utilize a restricted range of PSs, including xylan, starch, glycogen, lichenan, and laminarin (the latter two only demonstrated in *Acidilobus*) (19–21). Meanwhile, metagenomics has revealed the greatest diversity and abundance of genes encoding carbohydrate-active enzymes (CAZymes) in uncultured archaeal lineages in geothermal environments, namely *Candidatus* Brockarchaeota, *Ca*. Geoarchaeota, and *Ca*. Marsarchaeota (22). The latter was detected in high abundance in hot acidic microbial mats, sediments, and thermal soils (9, 23) and, based on various omics analyses, it was suggested that this group is represented by thermophilic facultative aerobic chemoheterotrophs that may utilize Fe^3+^ as a terminal electron acceptor under low O_2_ conditions (23). To date, no representatives of *Ca*. Marsarchaeota have been isolated in pure cultures, so their properties remain only hypothetical.

In this work, we studied the phenotypic features and performed a detailed genomic analysis of the first cultivated representatives of the group *Ca*. Marsarchaeota, strains MP-3918 and AK-3817. We revealed that their metabolism differs significantly from previously predicted, as well as from all thermoacidophilic prokaryotes known to date. Novel archaea species conserve energy only through fermentation on carbohydrates, especially PSs. Genome analysis allowed us to clarify their taxonomy and to conduct the comparative analysis of the new order *Tardisphaerales,* which occupies a significant part in the microbial communities of acidic hot springs.

## RESULTS

### Enrichment and isolation

Culture MP-3918 was enriched in anaerobic medium supplemented with starch and sulfur at pH 4.2 and 54°C during 100 days of incubation in Hungate tubes. The target group made up 80% of the obtained primary enrichment community (Fig. S1). The target group appeared in the second transfer only after 45 days of incubation and further increased continuously to 99% at 113 days. Strain MP-3918 was obtained only after varying the polysaccharide substrates (starch, karaya gum, pectin, or pullulan), and a pure culture was isolated after two additional successive transfers on pullulan, each lasting 100 days. Strain AK-3817 was enriched over a series of subsequent passages with prolonged incubation (each passage was incubated for at least 1 month) on anaerobic medium (at pH 4.0), containing glucose as the sole source of carbon and energy, supplemented with sulfur, at 65°C. To inhibit bacterial contaminants, vancomycin (40 mg L^−1^) was added. Furthermore, the culture was subjected to long-term incubation (1–2 months) in sulfur-free medium with glucose (or with pullulan, or starch) at 70°C with novobiocin (40 mg L^−1^) to suppress archaeal satellites (e.g., *Thermoplasma* spp.). The pure culture of strain AK-3817 was obtained using the serial dilution-to-extinction method on the medium with pullulan at pH 4.0 at 70°C.

### Phenotypic characteristics and metabolism

**Fig 1:**
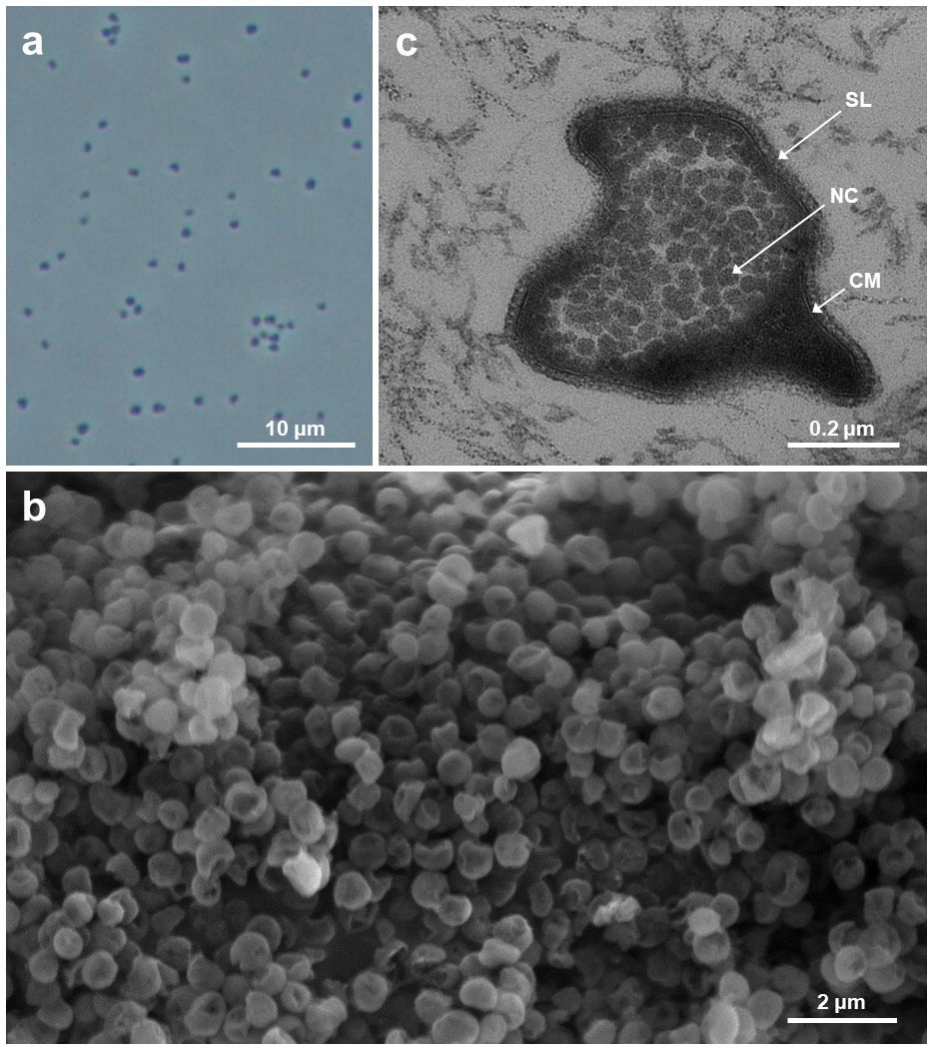
Morphology of MP-3918 (**a and b**) and AK-3817 (**c**) cells. (**a**) An image of phase-contrast microscopy. (**b**) An image of scanning electron microscopy. (**c**) An image of ultrathin-section transmission electron microscopy. S-layer (SL), cell membrane (CM), and nanocompartments (NC).

Cells of strains MP-3918 and AK-3817 were small irregular cocci 0.4–1.0 μm in diameter (Fig. 1; Fig. S2), surrounded by a thick S-layer (~20 nm) found to be arranged in a regular lattice with p4 symmetry (Fig. S2a). A single pili-like surface appendage 10–12 nm in diameter was observed (Fig. S2c). Under the electron microscope, cells often looked deflated, increasing the surface area to volume ratio. Intracellular compartments (35–40 nm in diameter) resembling encapsulin-covered nanocompartments were observed (24). The major intact polar lipids in both strains were a range of diphytanyl glycerol diethers (archaeols) and a range of membrane-spanning, glycerol dialkyl glycerol tetraethers (GDGTs), with 0–4 cyclopentane moieties (Table S1). No archaeal quinones (25) were detected in either strain, possibly as they were under the limit of analytical detection.

Both strains were thermoacidophiles, growing optimally at 55°C–65°C and at pH 3.9–4.0, anaerobic chemoorganoheterotrophs, able to ferment carbohydrates, especially PSs (Table S2). Neither auto-, lithotrophic growth (with H_2_/sulfur/Fe^2+^ as electron donors) nor respiration (with O_2_/sulfur compounds/Fe^3+^/NO_3_^-^/NO_2_^-^ as electron acceptors) was observed. Sulfur stimulated the cell yield of both strains, but the growth was shown even in the absence of sulfur. Growth curves revealed t_d_ of 52–91 hours in the exponential phase. The maximal cell yield was ~0.8–3 × 10^8^ cells mL^−1^ and strains kept high cell densities up to 1.5 months of incubation in periodic culture (Fig. S3a). The main growth products on carbohydrates were acetate and CO_2_, also ethanol and *n*-butyrate were detected in trace quantities, and no H_2_ was produced. H_2_S was observed in the case of cultivation with sulfur in trace quantities (Fig. S3b). No cell lysis was observed in culture MP-3918 during the 52-day incubation period under an oxygen-containing atmosphere (Fig. S3c). Upon transfer to fresh anaerobic medium, the cells resumed growth and reached the expected density.

### Genome characteristics and phylogenetic analysis

The genome of the strain MP-3918 was assembled into a circular chromosome with a size of 1,429,880 bp and a G + C content of 53.6%, while the genome of the strain AK-3817 consisted of 12 scaffolds with a size of 1,498,369 bp and a G + C content of 51.9% (Fig. S4; Table S3). The latter was almost complete and of equally high quality, with theoretical completeness 91.36% (compared to 92.13% for the MP-3918 complete genome), and contamination level 0.93% (for both genomes). According to the NCBI Prokaryotic Genome Annotation Pipeline (PGAP), the genomes of strains MP-3918/AK-3817 contained 1,361/1,382 protein-coding genes, a single rRNA operon, 46 tRNA genes, 2 ncRNA genes, and 24/29 pseudogenes, respectively.

**Fig 2:**
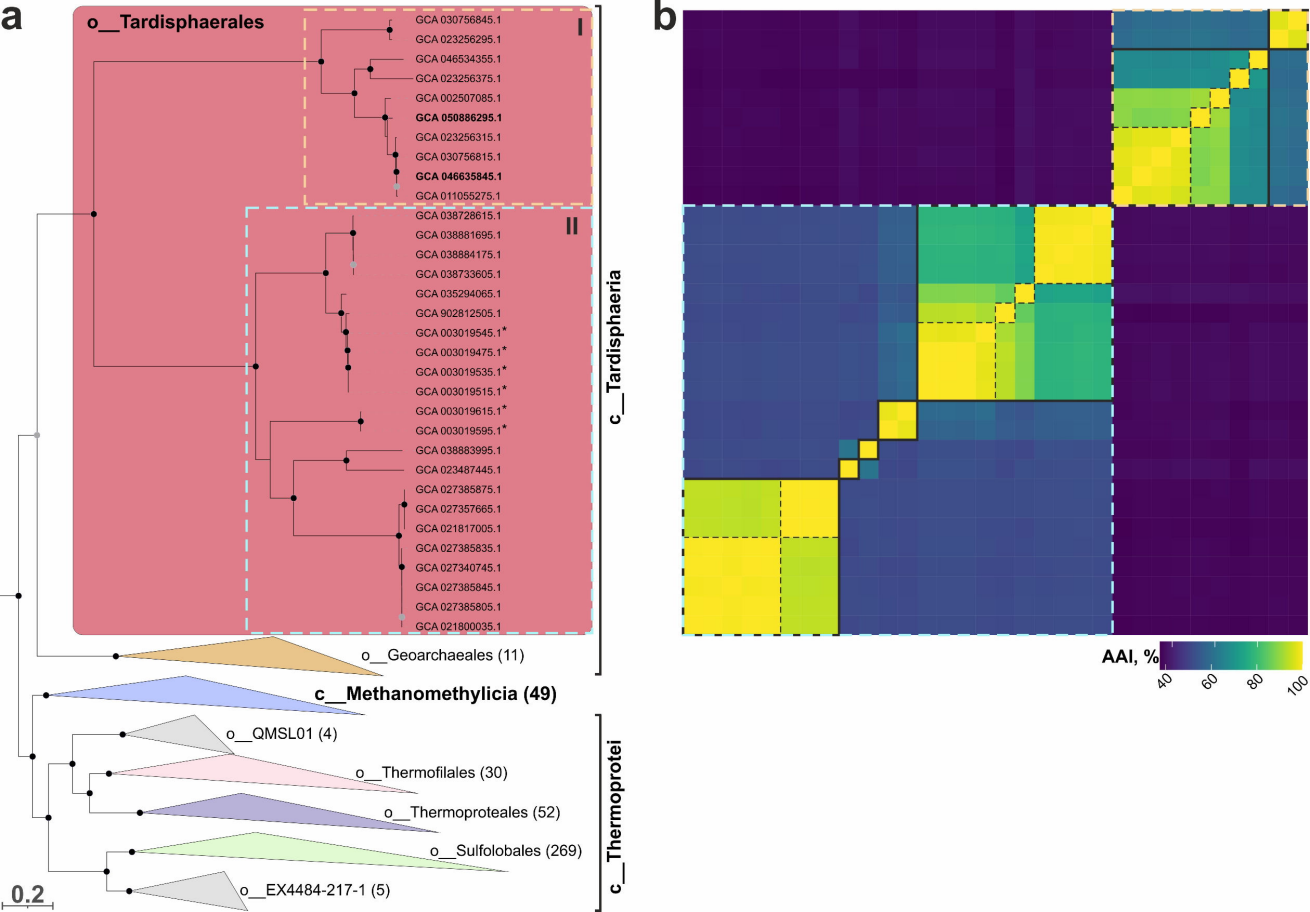
(**a**) Phylogenomic subtree based on the “ar53” protein set (full tree is presented in Supplementary material as Fig. S5 and as File S1 in tree format), which revealed the position of the strains MP-3918 and AK-3817 (in bold) within related taxa of the *Thermoproteota* phylum. Genomes previously analyzed by Jay et al. (23) are marked with asterisks. The proposed novel order *Tardisphaerales* is divided into two families, I*—Tardisphaeraceae* and II*—Ca*. Martarchaeaceae. The black circles at nodes indicate the percentage values of ultrafast bootstrap test (from 1,000 replicates) are higher than 95%, while gray circles—between 90% and 94%. (**b**) Matrix of AAI values between representatives of *Tardisphaerales*. Genomes fall in the same order as on the phylogenetic tree. Representatives of *Tardisphaeraceae* are bordered with a pastel yellow dotted line, while members of *Ca*. Martarchaeaceae—by light blue dotted line. Separate genera and species are bordered with black bold lines and black dotted lines, respectively.

BLAST analysis of the 16S rRNA genes showed that the closest relatives were environmental archaeal clones from the hot springs of Japan, Taiwan, and Russia with 99.5%–99.9% sequence identities. In contrast, the closest cultivated species belonged to the class *Thermoprotei* with identities <85%, indicating that the isolates represent a novel deep-branching lineage. To identify the exact phylogenetic position of the strains, a phylogenomic analysis based on the “ar53” marker set was performed. This analysis revealed that the strains fell into the clade within the phylum *Thermoproteota* (Fig. S5). In addition to our strains, this clade contained archaea previously described as *Ca*. Marsarchaeota representatives (23). However, according to our phylogenetic analysis, this group formed an order-level lineage (proposed name o__Tardisphaerales) which together with o__Gearchaeales forms a single class within the *Thermoproteota* phylum (Fig. 2a; File S1). Two other relatively close classes are c__Methanomethylicia (should be named as *Ca*. Methanomethylicia) and c__Thermoprotei; the latter contains the orders with cultivated species o__Sulfolobales, o__Thermoproteales, o__Thermofilales and several small orders without cultivated members. This classification is supported by both the tree topology and the relative evolutionary divergence (RED). The RED values for the proposed novel class c__Tardisphaeria, c__Methanomethylicia, and c__Thermoprotei were 0.32, 0.337, and 0.329, respectively (File S1). These values are relatively close to the median for classes calculated from our tree –0.391 (Table S4). An alternative version is merging all clades shown in Fig. 2a into a single large class. However, the RED value for this node is 0.273 (File S1), which is significantly below the median. Therefore, this alternative classification seems incorrect.

The novel order should be divided into two families: f__Tardisphaeraceae, which includes our isolates, and f__Martarchaeaceae with metagenome-assembled genomes (MAGs) analyzed earlier by Jay and colleagues (23). The RED value for f__Tardisphaeraceae was 0.857, while that for f__Martarchaeaceae was 0.756 (File S1). Although the RED values are slightly higher than the median for the family level (0.72), we propose that such family classification is optimal because the deeper node had the RED value of 0.436. Based on a comparison of average amino acid identity (AAI) values (considering the threshold for the genus and for the species of 65% and 95%, respectively [26]), two genera were predicted within f__Tardisphaeraceae (Fig. 2b; Fig. S6). One of these, with the proposed name *Tardisphaera*, is divided into five species, including two species represented by the strains MP-3918 (*Tardisphaera miroshnichenkoaea*) and AK-3817 (*Tardisphaera saccharovorans*). The second family, f__Martarchaeaceae, includes five genera (1–4 species in each genus).

### Genomes analysis

**Fig 3:**
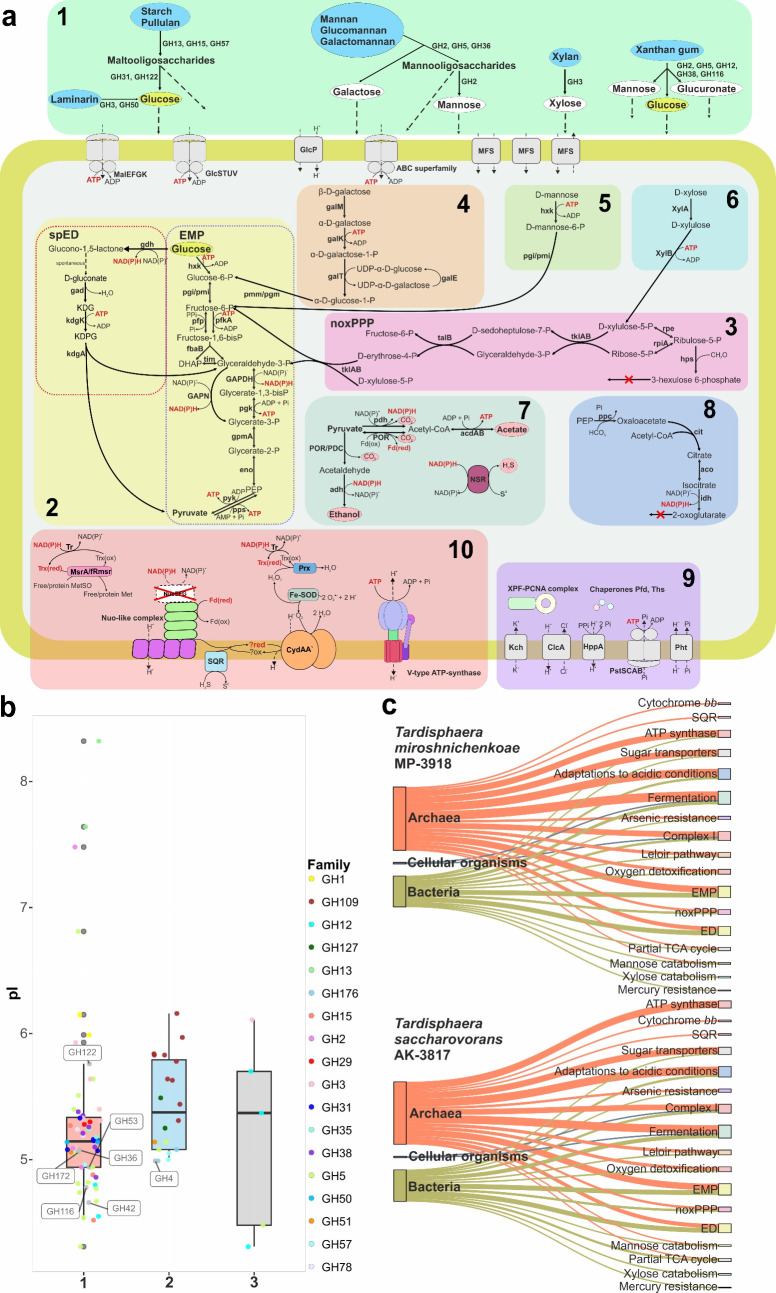
Metabolism features of the genus *Tardisphaera*. (**a**) Schematic map of *Tardisphaera* representative metabolism. 1—polysaccharides degradation, 2—glucose catabolism, 3—non-oxidative branch of PPP, 4—galactose catabolism, 5—mannose catabolism, 6—xylose catabolism, 7—fermentation, 8—partial TCA cycle, 9—acidophilic adaptations, and 10—ROS detoxification system. Deciphering of all enzyme abbreviations is given in Table S5. Red crosses indicate: Nuo-like complex lacks EFG subunits and presumably oxidizes ferredoxins but not NADH; TCA cycle is partial due to the absence of iso-citrate dehydrogenase and other enzymes; the genomes of strains do not contain genes of 3-hexulose-6-phosphate synthase for formaldehyde detoxification known for some archaea. (**b**) Distribution of isoelectric points of glycoside hydrolases (GHs): 1—extracellular glycosidases (including those lacking a signal peptide but predicted to be secreted by deepTMHMM) encoded in *Tardisphaera* genomes, 2—intracellular glycosidases encoded in *Tardisphaera* genomes, 3—characterized acidophilic glycosidases, GH3 family enzyme from *Thermofilum pendens* (WP_011753156.1), GH5 family enzyme obtained from metagenome screening (PQ498720) and three GH12 family enzymes from *Saccharolobus solfataricus*, *Acidothermus cellulolyticus*, and *Aspergillus niger* (Q97 × 08, A0LSI2, O74705). (**c**) Possible horizontal gene transfers from *Bacteria* to *T. miroshnichenkoae* and *T. saccharovorans* based on the HGTector 2 results.

The genomic characteristics of the isolates reflected their acidophilic lifestyle. Both genomes encode a single system for K^+^ uptake, voltage-gated channel Kch (Fig. 3a), presumably providing internal positive membrane potential. While genes for other potassium transporters, Trk, Kdp, Kup, as well as K^+^/H^+^ antiporters (27) were not found. Proton efflux could occur through the action of a pyrophosphate-energized pump HppA and a Cl^−^/H^+^ antiporter ClcA. Genes encoding the inorganic phosphate ABC transporter PstSCAB and the symporter Pht participating in buffering of protons in the cytoplasm were detected (28). Genes of urease, which provide buffering capacity, and glutamate or arginine decarboxylases, which catalyze proton consumption (29), were absent. Nuclease XPF and chaperones, which are responsible for DNA repair and correct protein folding under aggressive acidic conditions (30), were encoded in the genomes of the isolates.

Metabolic features deduced from the genome analysis were consistent with the physiological characterization. *Tardisphaera* genomes did not possess genes of the key enzymes for CO_2_ fixation pathways (31). Meanwhile, they obtained numerous genes encoding CAZymes (Table S6): 38 and 44 glycoside hydrolases (GHs) and carbohydrate esterases (CEs) were encoded in the genomes of MP-3918 and AK-3817, respectively. Glycosyltransferase diversity and functions were not in focus of the study, and they were excluded from the analysis. It should be noted that all of the predicted activities of the CAZymes are putative because they are based on genome analysis only. Both strains can grow on dextrin, starch, and pullulan due to the action of alpha-amylases (GH57), neopullulanase (GH13), glucoamylase (GH15), and alpha-glucosidases (GH122 and/or GH31). Xanthan gum decomposition pathway includes potential alpha-mannosidase (GH38), beta-glucuronidase (GH2), beta-mannosidase (GH2, found only in AK-3817 strain), some endoglucanases (GH5 or GH12 families), and beta-glucosidases from GH116 and/or GH3. Beta-glucan and laminarin are utilized by both strains due to the action of beta-glucosidases and endo-beta-1,3-glucanase from GH50. Xylan can be partially hydrolyzed by beta-xylosidases from the GH3 family, while endo-beta-1,4-xylanase genes were not found. Growth with mannan and galactomannan was observed for the studied strains; endo-beta-mannosidases from GH5 and alpha-galactosidase (GH4 or GH36 family) participate in mannan degradation; however, beta-mannosidase was encoded only in the AK-3817 genome. GHs predicted as extracellular enzymes possessed the low median isoelectric point (pI) of 5.15, which is close to the values of pI for known characterized acidophilic glycosidases (Fig. 3b). Furthermore, even intracellular glycosidases of the novel isolates showed the pI values less than 6. Oligo- or monosaccharides could be imported into the cells by ABC transporters and different porters, while phosphotransferase transport systems were not encoded in the genomes. All genes of the EMP and semi-phosphorylative ED (spED) pathways were found in *Tardisphaera* genomes (Fig. 3a; Table S5). Both strains possessed the Leloir pathway for galactose utilization (32). Xylose could be converted to xylulose-5-phosphate, which is next metabolized in non-oxidative PPP (noxPPP).

Genome analysis did not reveal genes encoding proteins of the complete electron transfer chains (ETC), required for aerobic or anaerobic respiration. Genes encoding multiheme cytochromes involved in iron reduction (33) were absent in both genomes, as well as genes of complex iron-sulfur molybdoprotein family oxidoreductases and cytochrome oxidases, also involved in anaerobic and aerobic respiration, respectively. However, genes encoding the terminal quinol oxidase CydAA were detected (Fig. 3a), even though its primary predicted function was not associated with proton-motive force generation (34). The TCA cycle was incomplete. Acetate could be formed from acetyl-CoA under the action of a two-subunit ADP-dependent acetate–CoA ligase. Two bifunctional pyruvate oxidoreductase complexes are presumably responsible for acetaldehyde formation (35), since no genes for acetaldehyde dehydrogenases or pyruvate decarboxylases were found in the genomes. Some of the numerous alcohol dehydrogenases could reduce acetaldehyde to ethanol during fermentation. Despite the presence of butyrate among the fermentation products, enzymes of known butyrate formation pathways were not encoded in the genomes. A gene encoding CoA-dependent NAD(P)H sulfur oxidoreductase (NSR) was detected in both genomes. Furthermore, the presence of sulfur stimulated growth of the strains, suggesting the possibility of “facilitated” fermentation (36), which led to H_2_S accumulation in the medium. Genome analysis did not reveal genes encoding [NiFe], [FeFe], or [Fe] hydrogenases, consistent with the observed lack of H_2_ production during fermentation.

Genome analysis revealed the presence of genes encoding iron superoxide dismutase (Fe-SOD), thioredoxin (Trx), thioredoxin reductase (TR), and several peroxiredoxins (Prx) in the genomes of both isolates. In addition, we found several genes, probably encoding rubrerythrins (Rub), distinguished by their domain organization from each other. Furthermore, genes of peptide methionine sulfoxide reductase A (MsrA) and free methionine-R-sulfoxide reductase (fRmsr) were identified in both genomes. In addition, cell protection from oxygen may be mediated by the CydAA membrane complex through oxygen reduction (34) with an unknown carrier as electron donor (since known quinones were not detected). Although genes encoding ferritin-like proteins were identified, the genomic analysis did not detect any encapsulin shell proteins, suggesting that the observed nanocompartments are structurally distinct from the canonical encapsulins (24).

Our analysis indicated that at least one-quarter of the genes involved in analyzed metabolic systems probably originated from the domain Bacteria and were acquired by *Tardisphaera* via horizontal gene transfer (HGT). Genes encoding enzymes for the initial steps of xylose catabolism (xylose isomerase, xylulose kinase), the noxPPP (ribulose-phosphate 3-epimerase, transketolase), and several glycolytic enzymes (phosphoglucose/phosphomannose isomerase, glyceraldehyde-3-phosphate dehydrogenase, phosphoglycerate mutase) are predicted to be of bacterial origin (Fig. 3c; Table S7).

### Comparative genomics and ecology of the order *Tardisphaerales*

**Fig 4:**
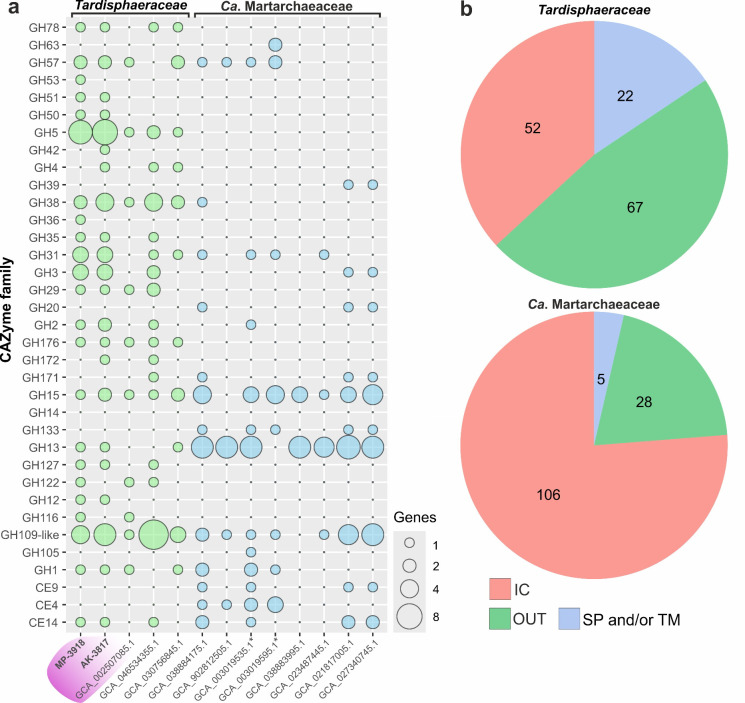
(**a**) GHs and CEs encoded in *Tardisphaerales* genomes. Only one genome per species is shown (full data are presented in Table S6). GH109-like glycosidases include GH109, GH177, and GH179 families, all of which possess the NAD^+^-dependent mechanism. Strains MP-3918 and AK-3817 are in bold; genomes affiliated with *Tardisphaera* genus are inside purple field; genomes previously analyzed by Jay et al. (23) are marked with asterisks. (**b**) Proportion of GHs and CEs which are predicted to be intracellular, extracellular, or membrane-bound among both families: IC—intracellular proteins, SP and/or TM—proteins with signal peptides and/or transmembrane regions, OUT—proteins without any signal peptides but are recognized as secreted outside the cell by DeepTMHMM. Analysis was based on representative genomes only (one genome per species).

Comparison of CAZymes encoded in both families of *Tardisphaerales* allowed the detection of signature differences (Fig. 4). Some CAZymes were represented by a higher number of genes in genomes of *Tardisphaeraceae* members (GH3, GH5, and GH31) or even their presence was unique for this family (GH12, GH29, GH35, GH78, GH122, GH127, and GH176). In contrast, most genomes of *Ca*. Martarchaeaceae were enriched with genes of enzymes from GH15 and GH13 families. Genes encoding some glycosidases (GH20, GH39, GH63, and GH133), carbohydrate esterases (CE4 and CE9), and carbohydrate oxidases (AA7) were found only in genomes of *Ca*. Martarchaeaceae members. Moreover, predicted enzyme profiles varied within the families: strains MP-3918, AK-3817, and MAG GCA_023256315.1 had a more vigorous CAZyme set than other *Tardisphaeraceae* members. Among *Ca*. Martarchaeaceae, these dissimilarities were less obvious. Most enzymes presumably possess only a single catalytic domain, and no proteins containing several catalytic domains from different CAZyme families were encoded in the analyzed genomes. However, there were some enzymes with two catalytic domains of the same family (e.g., GH122 + GH122) or with carbohydrate-binding modules (e.g., CBM67 + GH78 and CBM48 + GH13). Additionally, representatives of the two families differ in the proportion of extracellular CAZymes (Fig. 4b). Among the *Tardipshaeraceae* family, approximately 16% of CAZymes possessed a signal peptide and/or transmembrane region, and another 48% were predicted to be extracellular despite lacking a signal peptide. At the same time, only 4% and 20% of CAZymes fell into these groups in *Ca*. Martarchaeaceae, respectively.

**Fig 5:**
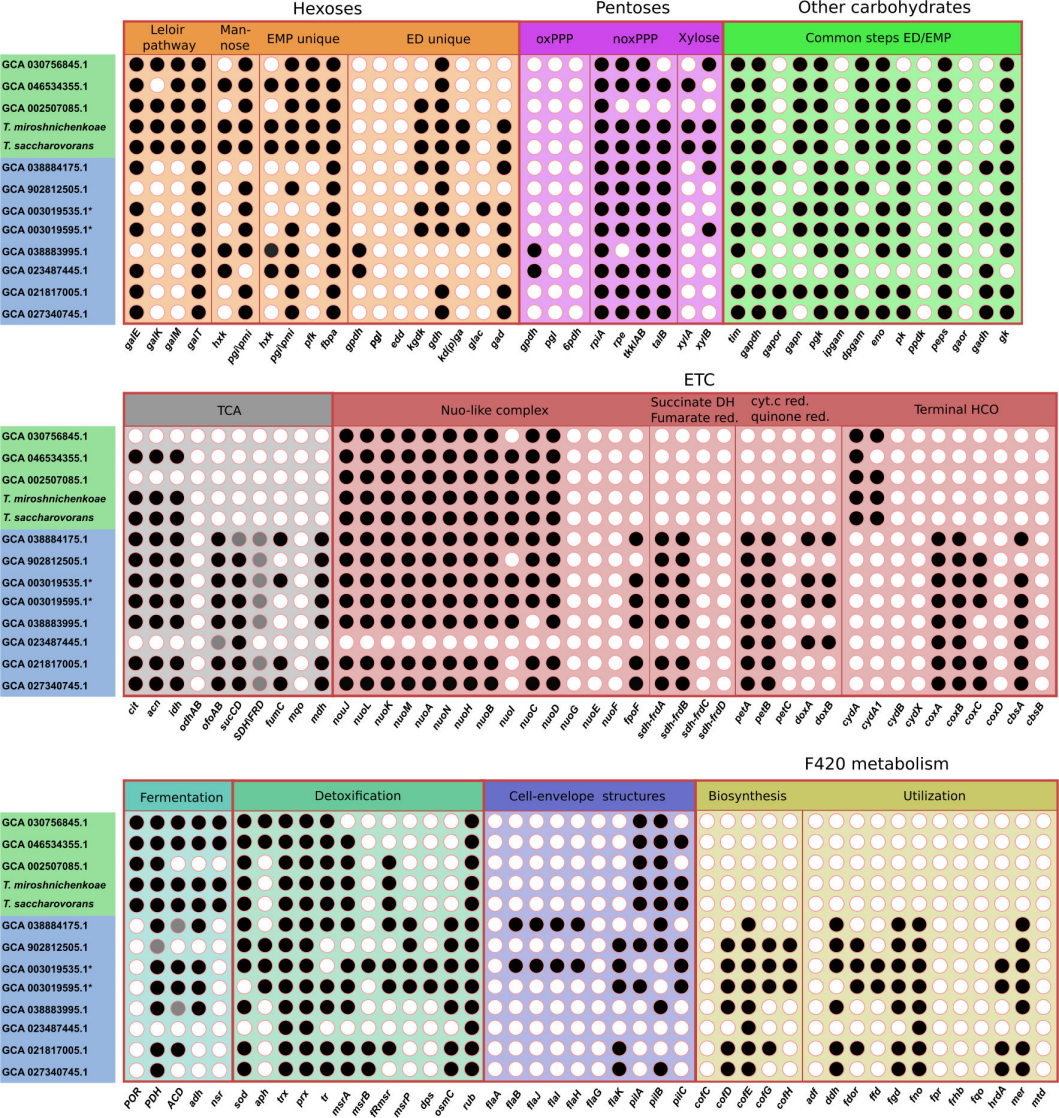
Comparative genomics of the order *Tardisphaerales* (only representative genomes are shown). The black dots indicate the presence of homologs in genomes, the gray dots—incomplete set of genes encoding protein complexes, the white dots—absence of homologs in genomes (Table S8). Organism names/genome numbers of *Tardisphaeraceae* and *Ca*. Martarchaeaceae families are within green and blue fields, respectively. Asterisk signs indicate MAGs described previously (23).

We performed a comparative genomic analysis of the central metabolic pathways identified in our strains, with the key metabolic enzymes predicted previously (23) in all available representatives of this order (Fig. 5). It revealed that all *Tardisphaeraceae* possess spED pathway, providing utilization of glucose, while *Ca*. Martarchaeaceae—both semi- and non-phosphorylated variants. The full EMP pathway was found only in members of *Tardisphaeraceae*. Homologs of all enzymes of the Leloir pathway were discovered in the *Tardisphaeraceae* family. In contrast, *Ca*. Martarchaeaceae representatives lack genes encoding galactokinase and galactose mutarotase and thus may not metabolize galactose. Homologs of the noxPPP enzymes were encoded in genomes of almost all *Tardisphaerales*. Xylose kinase and xylose isomerase were found only in *Tardisphaera* genomes, and xylose degradation could be the unique feature of the novel isolates among the order.

Genes encoding all enzymes of the TCA cycle were found only in genomes of *Ca*. Martarchaeaceae, while in *Tardisphaeraceae,* they were detected partially or completely absent. Several complexes of the aerobic ETC were encoded in MAGs from *Ca*. Martarchaeaceae. Eleven genes encoding subunits of Nuo-like complex were found in all *Tardisphaerales*; however, none of the genomes encoded the NuoE, NuoF, and NuoG subunits essential for NAD(P)H oxidation. Additionally, homologs of the *fpoF* gene, presumably allowing to use F_420_H_2_ as an electron donor in the ETC, were discovered in *Ca*. Martarchaeaceae, which agrees with the presence of F_420_ biosynthesis and metabolism genes in MAGs. Genomes of *Ca*. Martarchaeaceae contained genes encoding two subunits of succinate dehydrogenase/fumarate reductase, cytochrome *bc_1_* complex, and cytochrome *aa_3_* oxidase, which collectively may construct a complete ETC, allowing them to reduce oxygen and conserve more energy from organics decomposition. The presence of genes encoding DoxAD complex, presumably involved in the oxidation of thiosulfate, was earlier reported in *Ca*. Martarchaeaceae genomes (23). However, our analysis revealed that the genomes of some *Ca*. Martarchaeaceae, as well as all *Tardisphaeraceae* genomes, lacked these genes. It is noteworthy that genes of CydAA homologs were identified exclusively in *Tardisphaeraceae*. Some detoxification systems, like DNA protection during starvation protein (Dps), osmotically inducible protein C (OsmC), methionine sulfoxide reductase B (MsrB), and methionine sulfoxide reductase P (MsrP), were observed in *Ca*. Martarchaeaceae MAGs (12), but not in *Tardisphaeraceae* genome assemblies.

**Fig 6:**
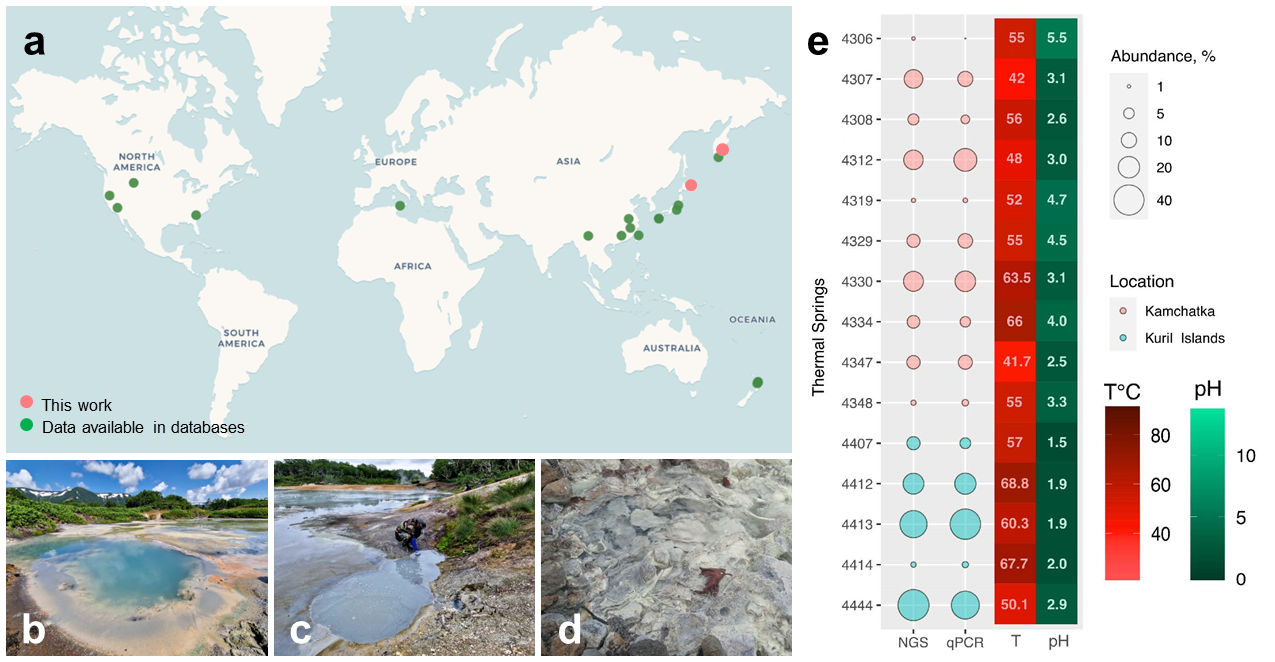
Ecological distribution of *Tardisphaerales* representatives and examples of hot spring sampling sites, where *Tardisphaerales* dominates. (**a**) Global distribution of *Tardisphaerales* in acidic environments based on 16S rRNA gene amplicon, clone library, and MAGs data sets. (**b**) Spring 4330 “The Beautiful.” (**c**) Spring 4307 “The Snowman” (both Kamchatka Peninsula, Uzon Caldera). (**d**) Spring 4444 (Kunashir Island, Mendeleev volcano). (**e**) Relative abundance of *Tardisphaerales* in thermal springs of Kamchatka and Kuril Islands according to NGS and qPCR. Temperature and pH correspond to parameters of the springs where *Tardisphaerales* were detected.

Screening of 16S rRNA gene amplicon, clone library, MAGs, and metagenomic reads data sets for sequences affiliated with representatives of *Tardisphaerales* order revealed their presence in samples from acidic hot springs, acid mine drainage (AMD), and reject coal pile sediments in Russia, Japan, China, Taiwan, New Zealand, Italy, and the United States (Fig. 6a). The acidity of those environments ranged from 1.5 to 5.5 and temperature—from 30 to 88°C (Table S9). To evaluate the abundance of the *Tardisphaerales* representatives in the environments, we performed qPCR screening in a broad range of Kuril-Kamchatka acidic hot springs, comparing the average abundance of all prokaryotes and the *Tardisphaerales* using universal and specific primers (Table S10). *Tardisphaerales* representatives were detected in the springs with temperatures ranging from 41.7°C to 69°C and pH of 1.5–5.5 (Fig. S7). The fractions of *Tardisphaerales* in microbial communities estimated by qPCR and NGS data were similar and constituted 0.8%–40.7%, with the highest values in hot springs with pH <3 (Fig. 6b through e).

### Description of *Tardisphaera miroshnichenkoae* and *Tardisphaera saccharovorans*

Based on the phylogenetic analyses and phenotypic properties, strains MP-3918 and AK-3817 represent a novel lineage within the *Thermoproteota*. We propose their classification as two novel species, *Tardisphaera miroshnichenkoae* sp. nov. (type strain MP-3918^T^ = VKM B-3629^T^ = CGMCC 1.18048^T^) and *Tardisphaera saccharovorans* sp. nov. (type strain AK-3817^T^ = VKM B-3679^T^ = CGMCC 1.18047^T^), within a new genus *Tardisphaera* gen. nov., which constitutes the type genus of *Tardisphaeraceae* fam. nov. and *Tardisphaerales* ord. nov. Together with the uncultivated *Ca*. Geoarchaeales, this order forms a distinct class within the phylum *Thermoproteota*, here proposed as *Tardisphaeria* class. nov.

(*Tardisphaera* gen. nov. [*Tar.di.sphae'ra*. L. masc. adj. *tardus,* slow; L. fem. n. *sphaera,* a sphere, a globe; N.L. fem. n. *Tardisphaera,* a slow-growing spherical organism]. Type species is *T. miroshnichenkoae*).

(*T. miroshnichenkoae* sp. nov. [mi.rosh.ni.chen’ko.ae; N.L. gen. fem. n. *miroshnichenkoae*, named in honor of Margarita Miroshnichenko, Russian microbiologist, for her contributions to microbial taxonomy and extremophile research]. Type strain is MP-3918^T^=VKM B-3629^T^=CGMCC 1.18048^T^, isolated from a solfatara on Kunashir Island [Kuril Islands]).

(*T. saccharovorans* sp. nov. [sac.cha.ro.vo’rans; N.L. neut. n. *saccharum*, sugar; L. pres. part. *vorans*, devouring; N.L. part. adj. *saccharovorans*, sugar-devouring]. Type strain is AK-3817^T^=VKM B-3679^T^=CGMCC 1.18047^T^, isolated from a hot spring in Uzon Caldera [Kamchatka]).

Both species are small irregular cocci (0.4–1 μm in diameter) with an S-layer and single pili-like appendage. Lipids are a mix of archaeol (19.5% in MP-3918, 43.3% in AK-3817) and GDGTs with 0–4 cyclopentane moieties. Strictly anaerobic. Thermoacidophiles. Growth occurs at pH 3.0–4.7 (optimum 3.9–4.0). *T. miroshnichenkoae* grows at 47°C–70°C (optimum 55°C–60°C), and *T. saccharovorans* at 37°C–75°C (optimum 65°C). Both ferment carbohydrates, with polysaccharides being the preferred substrates (see Table S2). *T. saccharovorans* additionally grows on yeast extract. Growth is stimulated by sulfur.

## DISCUSSION

With advances in genomic sequencing, the study of archaea in recent decades has brought us closer to the understanding of their importance for ecology, biotechnology, and evolution (37–39). Many deep phylogenetic lineages have been discovered by the progress in metagenomics, greatly expanding the understanding of the archaeal diversity (40–42). Clearly, as the volume of available genomic data continues to increase, the additional candidate archaeal lineages will be identified, and the insights into their metabolic functions and ecological roles will be derived through *in silico* analysis of MAGs. In turn, the investigation of pure (or stably enriched) cultures is a key source of reliable and high-quality data on the metabolism and phenotypic characteristics of organisms that cannot be completely seen through genomic analysis alone (38, 43).

Here, we described the metabolism of thermoacidophilic archaea—the first cultivated representatives of the deep phylogenetic lineage, assigned previously as *Ca*. Marsarchaeota (23, 44). Phylogenomic analysis of the two isolates, named *Tardisphaera miroshnichenkoae* and *Tardisphaera saccharovorans*, along with high-quality MAGs belonging to this group, demonstrated a distinct clustering of this lineage as the separate order (*Tardisphaerales*). Together with the uncultivated *Ca*. Geoarchaeales, this order defines a new class (*Tardisphaeria*) within the phylum *Thermoproteota* (Fig. 2). *Tardisphaerales* is divided into two families: the first one is *Ca*. Martarchaeaceae, including MAGs previously described and possessing presumably facultative anaerobic heterotrophs with a respiratory metabolism (23, our analysis), and the second one*—Tardisphaeraceae*, including the novel isolates.

*Tardisphaera* species conserve energy solely through fermentation, a metabolic strategy that, to our knowledge, is unique among all known thermoacidophilic organisms (13). The novel strains could perform “facilitated” fermentation (45) by the presence of NSR, which allows the use of elemental sulfur as an external acceptor during futile electron transfer and facilitates NAD^+^/NADH cycling in the cytoplasm (46). Moreover, the first isolated *Tardisphaerales* demonstrated notable resistance to acidic conditions previously not shown for thermoacidophiles. Strains maintained high abundance under pH 4.0 during 1–2 months in pure culture and more than half a year in highly enriched cultures. Hence, the membranes of the new strains are compositionally distinct from other thermoacidophilic archaea due to the significant proportion of archaeol (19%–43%). Lipids of moderate thermophilic *Tardisphaera* species rather resemble those of hyperthermophilic archaea in terms of the ratio of diethers/tetraethers and polar head groups structure (47). Genomic analysis revealed genes involved in adaptation to acidic conditions, like reverse membrane potential produced by K^+^ influx, proton-efflux defense, buffering of protons in cytoplasm, DNA, and protein repair systems (10, 48). Moreover, the strains exhibit remarkable tolerance to prolonged oxygen exposure, supported by one of the most robust ROS defense systems identified among thermoacidophilic organisms (12). It enables archaea to thrive in acidic habitats with elevated oxidative stress.

Genomic analysis identified a great variety of glycoside hydrolase genes in both strains (Fig. 4). Cultural experiments proved that they can metabolize a wide range of natural PSs, both soluble and insoluble, with alpha- and beta-linkages. Novel archaea are the first thermoacidophilic cultivars with such a broad ability to hydrolyze PSs. Comparative genomics of the *Tardisphaerales* revealed that carbohydrate metabolism is a defining feature of this order, though the two families differ markedly in their glycosidase repertoires, indicating distinct substrate preferences (Fig. 4). Notably, genes encoding specific chitinases were absent from the genomes, consistent with the lack of growth on chitin. Similarly, although potential endoglucanases (GH5 and GH12 families) were detected, the isolates did not grow on cellulose. Thus, these archaea appear to lack specialization for degrading the most abundant natural polysaccharides (chitin and cellulose), but instead show adaptations for utilizing a broader range of less common substrates. In line with this, the *Tardisphaeraceae* genomes encode a higher proportion of potentially extracellular CAZymes compared with *Ca*. Martarchaeaceae, indicating stronger specialization in polysaccharide degradation, while *Ca*. Martarchaeaceae likely utilize oligosaccharides or carbohydrate monomers.

Monosaccharides released during PS hydrolysis are further metabolized through the multiple central carbohydrate pathways, and genes encoding the complete enzymatic repertoire for these processes (the EMP, spED, and noxPPP pathways) were identified in *Tardisphaera* genomes (Fig. 3a). Some of these genes (such as those of the noxPPP and genes involved in xylose catabolism) appear to have been transferred from the domain Bacteria via HGT. In contrast, others, including those involved in fermentation pathways, adaptation to acidic conditions, and antioxidant systems, predominantly encode proteins of archaeal origin (Fig. 3c). The coexistence of multiple parallel sugar degradation pathways is rare among archaea but has been reported in several cases. For example, *S. solfataricus* uses both the modified ED pathway and a pentose degradation way leading to alpha-ketoglutarate, but preferentially metabolizes glucose, which represses arabinose uptake (49). The halophile *Halococcus saccharolyticus* catabolizes glucose via the ED and fructose via the EMP pathways, resulting in diauxic growth (50). *Thermoproteus tenax* employs both modified EMP and ED pathways, converting ~85% of glucose through glycolysis and ~15% through the branched ED pathway, as demonstrated by ^13^C-NMR studies (14). To our knowledge, it is the only archaeon known to use both pathways in parallel.

**Fig 7:**
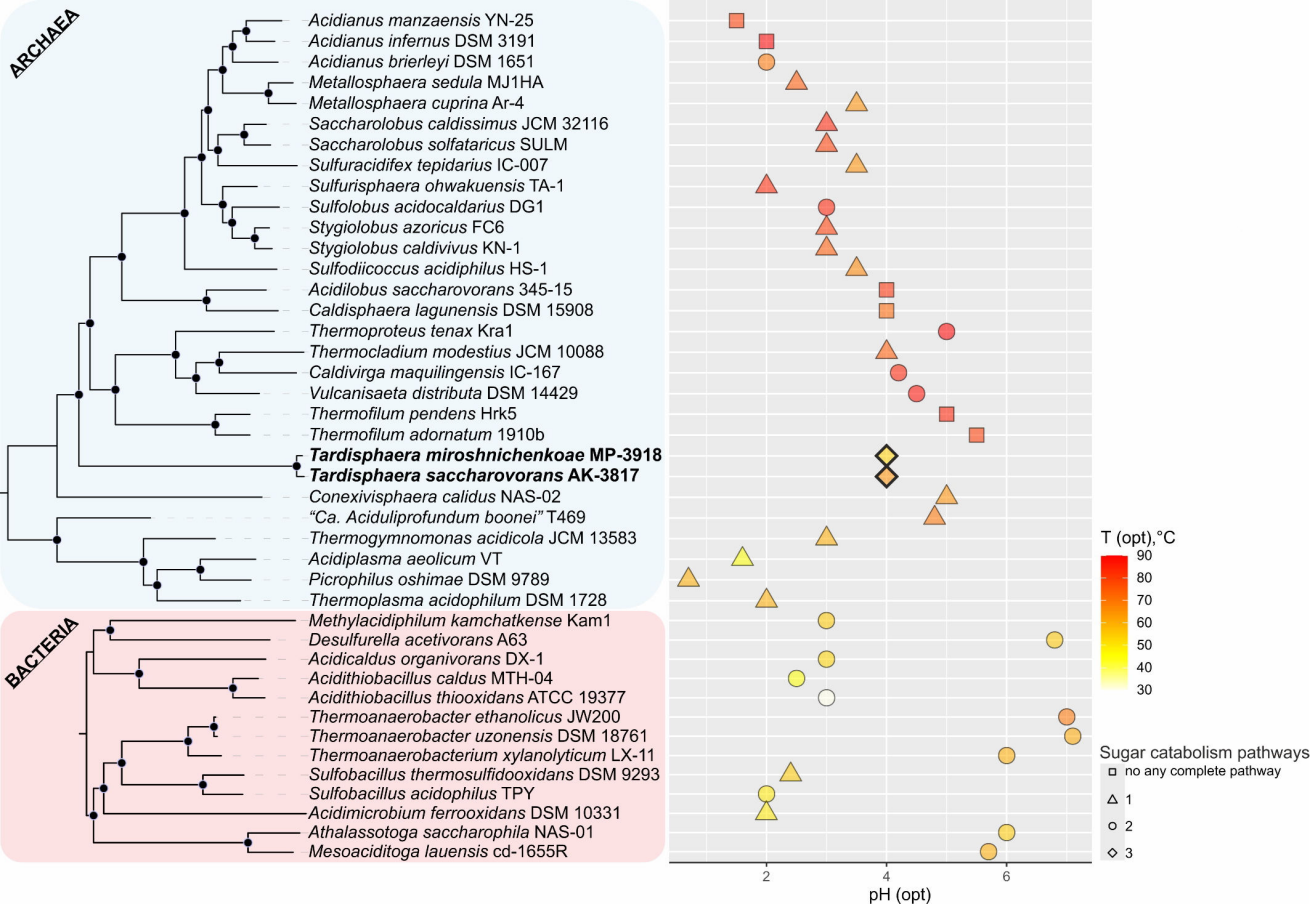
Sugar metabolism pathways (EMP, ED, and PPP) identified in genomes of cultivated thermoacidophilic and thermoacidotolerant prokaryotes.

Comparative genomic analysis of isolated heterotrophic thermoacidophilic and acidotolerant prokaryotes showed that *Tardisphaera* encodes the most diverse set of sugar degradation pathways among the analyzed taxa (Fig. 7; Table S11). Whether these pathways operate simultaneously remains unclear, and experimental confirmation is required. Intriguingly, despite the possessing of the enzymatic repertoire typically associated with complex carbohydrate degradation and metabolic flexibility, *Tardisphaera* genomes are streamlined: they are remarkably small (~1.4–1.5 Mbp), encode only one rRNA operon, contain few pseudogenes, and have a low proportion of non-coding DNA (51). This apparent paradox (metabolic versatility within compact genomes) likely reflects evolutionary streamlining. Together with their small coccoid cell size (high surface-to-volume ratio) and large natural population sizes, these features suggest adaptations for efficient resource use (52). Genomic streamlining is a well-recognized strategy in oligotrophic prokaryotes, which optimize the substrate affinity and energy yield to succeed in nutrient-poor environments (52–54). However, in *Tardisphaera*, this adaptation may also benefit the copiotrophic lifestyle in nutrient-rich habitats, such as hot spring sediments at moderately high temperatures (55). Thus, the unusual combination of genome reduction and metabolic flexibility in *Tardisphaera* likely enhances both energy conservation and substrate utilization across fluctuating environmental conditions, albeit largely via low-energy-yielding fermentative processes.

Furthermore, our results indicate that *Tardisphaerales* representatives inhabit acidic environments worldwide, including geothermal and mesophilic sites, associated with AMD and sulfur coal piles. Our precise quantitative analysis confirmed that *Tardisphaerales* were consistently abundant (comprising up to 40% of the microbial community) in acidic hot springs of the Kuril-Kamchatka Region with temperatures reaching 70°C (Fig. 6). This high prevalence suggests a key ecological role, likely as primary contributors to the initial degradation of organic matter in these extreme environments. We showed that *Tardisphaera* proteins with predicted extracellular carbohydrate depolymerizing activity, such as different endoglucanases, endo-beta-mannosidases, and alpha-amylases, have the low pI values, suggesting that these archaea can be considered as a source of diverse acid-stable thermozymes suitable for industrial processes requiring acidic conditions and high temperatures, for example, in the fields of green/white biotechnology, animal feed production, and starch industry (56–58).

In summary, we conducted detailed whole-genome analyses of the first isolated representatives of a deep archaeal phylogenetic lineage (formerly *Ca*. Marsarchaeota). Functional analysis, coupled with the microbiological experiments, revealed quite different features than had been previously predicted for this group based on metagenomics (23). The first archaea of the new order are moderately thermophilic and acidophilic organisms with small coccoid cells, growing anaerobically exclusively by fermentation on carbohydrates. A key distinguishing trait of *Tardisphaerales* compared to other thermoacidophilic prokaryotes is their extensive repertoire of enzymes specialized in hydrolyzing diverse natural polysaccharides. Their high abundance of CAZymes, likely stable and functional under low pH and high temperature conditions, makes the new isolates a plentiful repository for the discovery of biotechnologically relevant proteins. Remarkably, *Tardisphaera* encodes three divergent sugar catabolism pathways, a metabolic complexity never before observed in thermoacidophilic prokaryotes. The combination of an efficient ROS defense system, long-term acid stability, and the unique ability of *Tardisphaerales* archaea to utilize ubiquitous polysaccharides, derived from higher plants, algae, and prokaryotes, likely explains their dominance in sediments of acidic environments with moderate temperatures. Our findings underscore the necessity of combining comparative genomics (MAGs and isolate genomes) with experimental microbiology to reliably confirm functional predictions.

## MATERIALS AND METHODS

### Sampling, isolation, and cultivation

In August 2019, a sample of sand and soil (#3918) from a solfatara located at the Golovnin caldera at Kunashir Island (Kurils, Russia) was collected into sterile 50 mL falcon tube; a mix of geothermal water and gray sediments from а mud spring (#3817) at Uzon Caldera (Kamchatka, Russia) was collected into sterile 50  mL flask (Table S12), with care taken to exclude any air bubbles, sealed with rubber stoppers and aluminum caps. The samples were transported to the laboratory at +4°C and used for enrichment cultures. Unless otherwise indicated, the following anaerobic modified Pfennig medium (19) was used for enrichment, isolation, and cultivation, pH adjusted to 3.9–4.2. The medium was poured into 15 mL Hungate tubes or 50/100 mL glass flasks under a N_2_ or CO_2_ gas, reduced with 0.35 g L^−1^ Na_2_S*9H_2_O and supplemented with organic substrates (see below) and 50 mg L^−1^ of yeast extract. Elemental sulfur was added in some of the tubes. The growth temperature was tested in the range from 30°C to 80°C with 5°C intervals. pH supporting the growth was examined in the range from 2.7 to 5.5 using HEPES (2.7–5.0) and MES (5.0–5.5) buffers. Sugars, PSs, proteinaceous substances, alcohols, or organic acids (Table S2) were tested as organic substrates. To validate the ability to grow under the target conditions, triple passages were performed. NO_3_^−^ (10 mM), NO_2_^−^ (2 mM), ferric citrate (10 mM), and ferrihydrite (50 mM of Fe^3+^) were added to Na_2_S-free medium and elemental sulfur (1 g L^−1^), SO_3_^2−^, SO_4_^2−^, S_2_O_3_^2−^, or polysulfide (10 mM each) were added to Na_2_S-medium to be tested as electron acceptors. Autotrophic growth in anaerobic conditions was tested using H_2_-CO_2_ (10:90 or 50:50 or 80:20, vol/vol) in the gas phase with S^0^, SO_4_^2−^, ferrihydrite, NO_2_^−^, or NO_3_^−^ as electron acceptors or using N_2_-CO_2_ (80:20, vol/vol) with FeCl_2_ (10 mM final) as electron donor and NO_3_^−^ or S^0^ as electron acceptors.

The ability to grow anaerobically on Na_2_S-free medium was tested with starch; microaerobic (N_2_-O_2_, 98:2, vol/vol), and aerobic growth—with starch, pullulan, glucose, or acetate. Oxygen tolerance was tested on grown cultures (at the last exponential stage) with or without sulfur (each variant in six replicates). For this purpose, in three flasks of each variant, the gas phase was changed to air, and the rubber stoppers were pierced with a sterile needle for free air access. All variants were incubated further at optimal temperature for an extra 81 days. After 7, 15, 25, 35, 52, and 81 days of incubation, cells were counted and transferred to fresh anaerobic medium to examine the duplication capacity after the oxygen stress.

H_2_S formation was determined colorimetrically as described (19). H_2_, CO_2_ production, and organic growth products (volatile fatty acids and alcohols) were determined in four replicates using gas chromatography on GC 5000.2 (Chromatek, Russia) as described previously (59).

### Morphology

To prepare ultrathin sections, the cells were concentrated by centrifugation (9,000 × *g*, 30 min) and fixed as described previously (60). Ultrathin sections were examined under a JEM-1400 transmission electron microscope (JEOL, Tokyo, Japan) at an accelerating voltage of 80 keV. For scanning electron microscopy (SEM), samples were prepared as described (61) using a vacuum sputtering equipment JFC 1100 (Jeol, Japan). The SEM analysis was performed using a JSM-6510LV (JEOL, Japan) microscope.

### Lipids analysis

For lipid analysis, the strains were grown on standard anaerobic medium supplemented with sulfur and maltose (MP-3918) or galactomannan (AK-3817) at pH 3.9 and 60°C. Intact polar lipids (IPLs) were extracted from freeze-dried biomass using a modified Bligh-Dyer procedure (62). Briefly, the biomass was treated ultrasonically two times for 10 min with a solvent mixture of MeOH, dichloromethane, and phosphate buffer (2:1:0.8, vol:vol:vol). After sonication, the combined supernatants were phase-separated by adding additional dichloromethane and phosphate buffer to a final solvent ratio of 1:1:0.9 (vol:vol:vol). The organic phase containing the IPLs was collected, and the aqueous phase was re-extracted two times with dichloromethane. The residue was then re-extracted following the same procedure but starting with a solvent mix of MeOH, dichloromethane, and trichloroacetic acid, pH 2–3 (2:1:0.8, vol:vol:vol). Finally, the combined extract was dried under a stream of N_2_ gas. Before analysis, the extract was redissolved in a mixture of MeOH:dichloromethane (9:1, vol:vol). Subsequently, aliquots were filtered through 0.45 µm regenerated cellulose syringe filters (4 mm diameter; Grace Alltech, Deerfield, IL, US). Analysis was carried out using Ultra High-Pressure Liquid Chromatography-High Resolution Mass Spectrometry (UHPLC-HRMS) according to the reversed phase method (63) with modifications as per (62). We used an Agilent 1290 Infinity I UHPLC equipped with a temperature-controlled auto-injector and column oven, coupled to a Q Exactive Orbitrap MS with Ion Max source with heated electrospray ionization (ESI) probe (Thermo Fisher Scientific, Waltham, MA, United States). Separation was achieved on an Acquity BEH C18 column (2.1 × 150 mm, 1.7 µm; Waters Corporation, Milford, MA, United States) maintained at 30°C. The eluent composition was (A) MeOH/H_2_O/formic acid/14.8 M NH_3aq_ (85:15:0.12:0.04 [vol:vol]) and (B) isopropanol/MeOH/formic acid/14.8 M NH_3aq_ (50:50:0.12:0.04 [vol:vol]). The elution program was: 5% B for 3 min, followed by a linear gradient to 60% B at 12 min and then to 100% B at 50 min; this was maintained until 80 min. The flow rate was 0.2 mL min^−1^. Positive ion ESI settings were as follows: capillary temperature, 300°C; sheath gas (N_2_) pressure, 40 arbitrary units (AU); auxiliary gas (N_2_) pressure, 10 AU; spray voltage, 4.5 kV; probe heater temperature, 50°C; S-lens 70 V. Target lipids were analyzed with a mass range of *m/z* 350–2,000 (resolving power 70,000 ppm at *m/z* 200), followed by data-dependent MS^2^ (resolving power 17,500 ppm), in which the 10 most abundant masses in the mass spectrum were fragmented successively (stepped normalized collision energy 15, 22.5, and 30; isolation width, 1.0 *m/z*). The MS was calibrated within a mass accuracy range of 1 ppm using the Thermo Scientific Pierce LTQ Velos ESI Positive Ion Calibration Solution. During analysis, dynamic exclusion was used to temporarily exclude masses (for 6 s) to allow selection of less abundant ions for MS^2^. IPLs were quantified in terms of their MS peak area response. As different IPLs show different response behavior, the relative abundance of peak area does not necessarily reflect the actual relative abundance of the different IPLs. The peak areas were determined from extracted ion chromatograms of the combined (M + H)^+^, (M+NH_4_)^+^, and (M + Na)^+^ ions, and their second isotope peaks (where present) for each individual IPL species.

### DNA extraction and 16S rRNA gene-based profiling

DNA was isolated using FastDNA Spin Kit for Soil (MP Biomedicals, USA) according to the manufacturer’s protocols. Cells of enrichment and pure cultures were harvested by centrifugation at +4°C, and DNA was extracted immediately. Library preparation protocol to sequence V4 region of 16S rRNA gene, sequencing, and analysis were performed as described previously (64). Sequencing was carried out on a MiSeq system (Illumina, San Diego, CA, USA) using a reagent kit, which can read 150 nucleotides from each end. Each sample was sequenced in two replicates.

### qPCR-based quantification

For qPCR-based total prokaryotes quantification, a pair of universal prokaryote primers was used: 515F (5′-GTGBCAGCMGCCGCGGTAA-3′) (65) and Pro-mod-805R (5′-GACTACNVGGGTMTCTAATCC-3′) (66). For quantification of *Tardisphaerales* representatives, a specific primer system was designed in Primrose software (67) using sequences of 16S rRNA gene of 29 *Tardisphaerales* including strains MP-3918 and AK-3817 isolated in the course of this work as well as 127 non-target sequences from other archaea as *in silico* negative controls (Table S13). The specificity of the designed primer system was verified by TestPrime 1.0 software (68). For calibration curves, the genomic DNA of strain MP-3918 was used. All qPCR runs, including standard curve, experimental samples, and negative controls, were performed in triplicates, and the mean quantities of 16S rRNA gene copies were calculated. Genomic DNA from two pure cultures of *Fervidicoccus fontis* strain 3639Fd and *Thermoplasma* strain 3817c were used as templates for negative controls. qPCR analyses were carried out on a StepOnePlus Real-Time PCR System (Thermo Fisher Scientific, USA) using the qPCRmix-HS SYBR kit (Evrogen, Russia). Relative abundance of *Tardisphaerales* based on qPCR data was calculated as the ratio between 16S rRNA gene copies of *Tardisphaerales* per mL of each sample and total prokaryotic 16S rRNA gene copies per mL of the same sample.

### Search for 16S rRNA gene sequences and MAGs of *Tardisphaerales* representatives across public databases

To evaluate the ecological distribution of *Tardisphaerales* representatives, several approaches were used. First, our own data from 16S rRNA gene amplicon sequencing of hot springs of Kamchatka and Kuril Islands, as well as the data sets available in ENA (https://www.ebi.ac.uk/ena/browser/home), were analyzed as described earlier (9). Additionally, homologs of 16S rRNA gene sequences of strains MP-3918 and AK-3817 were found using BLASTn search against the NCBI core_nt database (https://blast.ncbi.nlm.nih.gov/Blast.cgi) and the Silva 138.2 database (https://ngs.arb-silva.de/silvangs/). Only BLAST hits with sequence identity >90% were taken into analysis (Table S9). Finally, isolation sources of MAGs affiliated with *Tardisphaerales* (o__Marsarchaeales in GTDB) according to GTDB r220 and metagenomic read data (with a threshold value of cANI > 0.95) available in Branchwater Metagenome Query (https://branchwater.jgi.doe.gov) were analyzed. The geographical location of samples, from which the data sets containing matching sequences were obtained, was visualized on a map using kepler.gl (https://kepler.gl).

### Genome sequencing and assembly

Genomic DNA from strains MP-3918 and AK-3817 was extracted using Genomic tip 20/G (Qiagen) and DNeasy PowerLyzer Microbial Kit (Qiagen), respectively. Libraries for Illumina sequencing were prepared using the DNA Prep kit (Illumina) and the KAPA Hyper Plus kit (KAPA) for strains MP-3918 and AK-3817. Library preparation for nanopore sequencing of the strain MP-3918 genome was done with Rapid Barcoding Kit (Oxford Nanopore Technologies). Illumina sequencing of genomes of strains MP-3918 and AK-3817 was performed using 2 × 250 bp and 2 × 100 bp paired-end reads and Novaseq 6000 platform (Illumina). Nanopore sequencing of the strain MP-3918 genome was done using GridION (ONT) with flow cell FLO-MIN106D (R9). Illumina reads were filtered by quality and length using CLC Genomics Workbench v.10 (Qiagen). The genome of the strain MP-3918 was assembled using Flye v.2.9 (69) with polishing using Pilon v.1.23 (70). The genome of strain AK-3817 was assembled using SPAdes v.3.15.4 (71) in –isolate mode and –trusted-contigs option (contigs from assembly obtained with Unicycler v.0.4.9 (72). Completeness and contamination levels of the assemblies were estimated using CheckM v.1.2.2 (73).

### Phylogenetic analysis and genome-based comparisons

Initial identification of the isolated strains was performed using a BLAST search with 16S rRNA gene sequences against the NCBI core_nt database. Phylogenetic positions of strains MP-3918 and AK-3817 were analyzed using comparison of conserved proteins, “ar53” set (44). The proteins were detected in *in silico* translated genomes of novel isolates and high-quality genomes, affiliated to this group (the thresholds are described in the “Genome analysis” section) as well as representative genomes of the phylum *Thermoproteota* according to GTDB r.226 and aligned using GTDB-tk v.2.4.1 (74). Maximum likelihood phylogenetic tree was constructed using IQtree v.3.0.1 (75) with best model predicted by ModelFinder (LG + F + I + G4) and 1,000 ultrafast bootstrap replications (76). The tree was visualized and decorated using iTOL v.6 (77). For the relative evolutionary divergences (REDs) calculation, full “ar53”-based alignment of representative genomes available in GTDB r.226 (https://data.gtdb.ecogenomic.org/releases/release226/226.0/genomic_files_reps) was used for phylogenetic tree construction, which was done as described above. The REDs values were estimated using PhyloRank v.0.1.12 (https://github.com/dparks1134/PhyloRank) with the obtained tree and the taxonomy file, for which GTDB nomenclature was taken as the base with manual curation of taxa related to the novel isolates. AAI values were calculated using the aai.rb script (78) with blastp as the detection tool.

### Genome analysis

Genomes of the two studied isolates were annotated with PGAP v.6.4 (79). Comparative genome analysis included the genomes of the target group available in GTDB r226 with completeness >75% and contamination <10% as well (Table S14). CAZymes were searched using dbCAN v.4 (80) with HMMER (81) and Diamond (82) tools. The functions revealed by dbCAN proteins were further verified using BLASTp against Swiss-Prot/PDB databases. Isoelectric points of GHs were calculated using IPC v.2.0 (83). The presence of the signal peptides and transmembrane regions in the CAZymes was predicted using SignalP v.6 (84) and DeepTMHMM (85), respectively. Enzymes involved in central carbon metabolism and energy conservation pathways, detoxification systems, and surface structures biosynthesis were identified as follows: (i) BLAST search with sequences of respective characterized proteins as queries and proteomes of the studied microorganisms as databases (e-value threshold 1e-3), (ii) BLAST of positive hits against Swiss-Prot or PDB database for better prediction of their function followed by analysis of their domain organization and the gene context of the respective genes. Transporters were searched as follows: (i) all proteins with predicted transmembrane regions identified by DeepTMHMM (85) were aligned with the sequences from the TCDB database (86) using BLAST and (ii) predicted functions of hits were refined by BLAST against the Swiss-Prot database. To detect potential HGT from bacteria to *Tardisphaera* genomes, we used HGTector 2 (87) with a pre-built database (2023 release). To specifically target HGTs originating from the *Bacteria* domain (taxonomy id: 2), we set *Thermoproteota* (1783275) as the self-group and *Archaea* (2157) as the close group.

## Data Availability

Genome sequences of strains MP-3918 and AK-3817 are available in GenBank under accession numbers CP117708.1 and JAQQXN000000000.1, respectively, within BioProject PRJNA931120.

## SUPPLEMENTAL MATERIAL

File S1 msystems.00710-25-s0001.svg Phylogenomic tree based on full "ar53"-based alignment of representative genomes available in GTDB r.226.

Supplemental material msystems.00710-25-s0002.pdf Supplemental figures and tables.

Collection Cetrificates msystems.00710-25-s0003.pdf Collection certificates of strain MP-3918 and AK-3817 deposition.

Table S5 msystems.00710-25-s0004.xlsx Genetic determinants of distinct metabolic features of strains MP-3918 and AK-3817.

Table S6 msystems.00710-25-s0005.xlsx CAZymes encoded in genomes of Tardisphaerales representatives.

Table S7 msystems.00710-25-s0006.xlsx HGTector 2 analysis for prediction Tardisphaera proteins origin and probable horizontal gene transfers.

Table S8 msystems.00710-25-s0007.xlsx Metabolic potential comparison among Tardisphaerales representatives.

Table S9 msystems.00710-25-s0008.xlsx 16S rRNA gene amplicon data sets, clone library sequences, and MAGs affiliated with Tardisphaerales sequences.

Table S11 msystems.00710-25-s0009.xlsx Genes encoding proteins involved in catabolism of glucose and pentoses found in the genomes of thermoacidophilic and thermoaciditolerant prokaryotes.

ASM does not own the copyrights to Supplemental Material that may be linked to, or accessed through, an article. The authors have granted ASM a non-exclusive, world-wide license to publish the Supplemental Material files. Please contact the corresponding author directly for reuse.
