# Supplementary material for: Polysaccharide-degrading archaea dominate acidic hot springs: genomic and cultivation insights into a novel Thermoproteota lineage
Source: mSystems. 2025 Sep 22;10(10):e00710-25. doi: 10.1128/msystems.00710-25 (PMC12542729; doi:10.1128/msystems.00710-25)
Supplement: Supplemental material — Supplemental figures and tables. [file msystems.00710-25-s0002.pdf]

Supplementary Materials

Polysaccharide-degrading archaea dominate acidic hot springs: genomic and cultivation insights into a novel *Thermoproteota* lineage

Maria I. Prokofeva[1†], Alina I. Karaseva[1,2†], Adolf S. Tulenkov[1,2], Alexandra A. Klyukina[1], Natalia E. Suzina[3], Nicole J. Bale[4], Anchelique Mets[4], Christa Schleper[5], Alexander G. Elcheninov[1‡], Tatiana V. Kochetkova[1‡#]

[†]Maria I. Prokofeva and Alina I. Karaseva are co-first authors.

[‡]Alexander G. Elcheninov and Tatiana V. Kochetkova are co-last authors.

[#]Correspondence: kochetkova.tatiana.v@gmail.com

[1] – Federal Research Centre “Fundamentals of Biotechnology” of the Russian Academy of Sciences, Moscow, Russia

[2] – Moscow Center for Advanced Studies, Moscow, Russia

[3] – G.K. Skryabin Institute of Biochemistry and Physiology of Microorganisms of the Russian Academy of Sciences, Federal Research Center “Pushchino Scientific Center for Biological Research of the Russian Academy of Sciences”, Pushchino, Moscow region, Russia

[4] – Department of Marine Microbiology and Biogeochemistry, NIOZ Royal Netherlands Institute for Sea Research, Den Burg, the Netherlands

[5] – Department of Functional and Evolutionary Ecology, Archaea Biology and Ecogenomics Unit, University of Vienna, Vienna, Austria

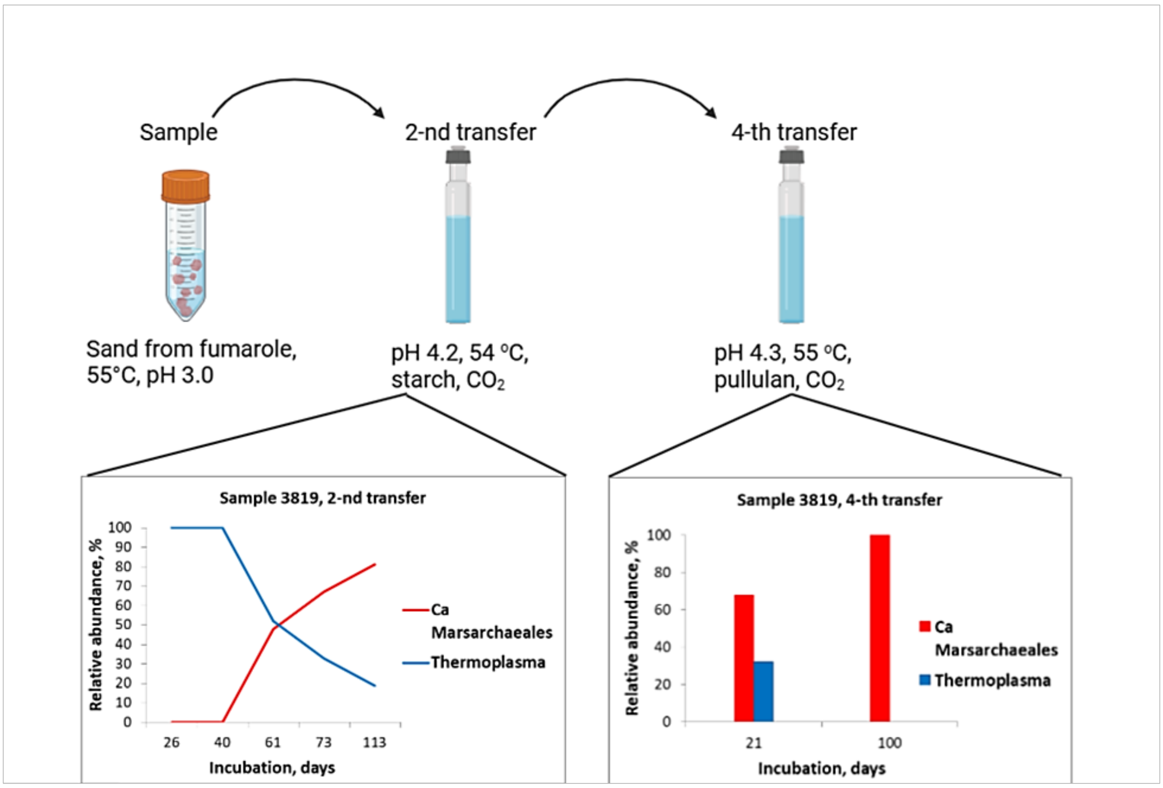

FIG S1 Changes in the community composition of the enrichment culture during incubation of sample 3918.

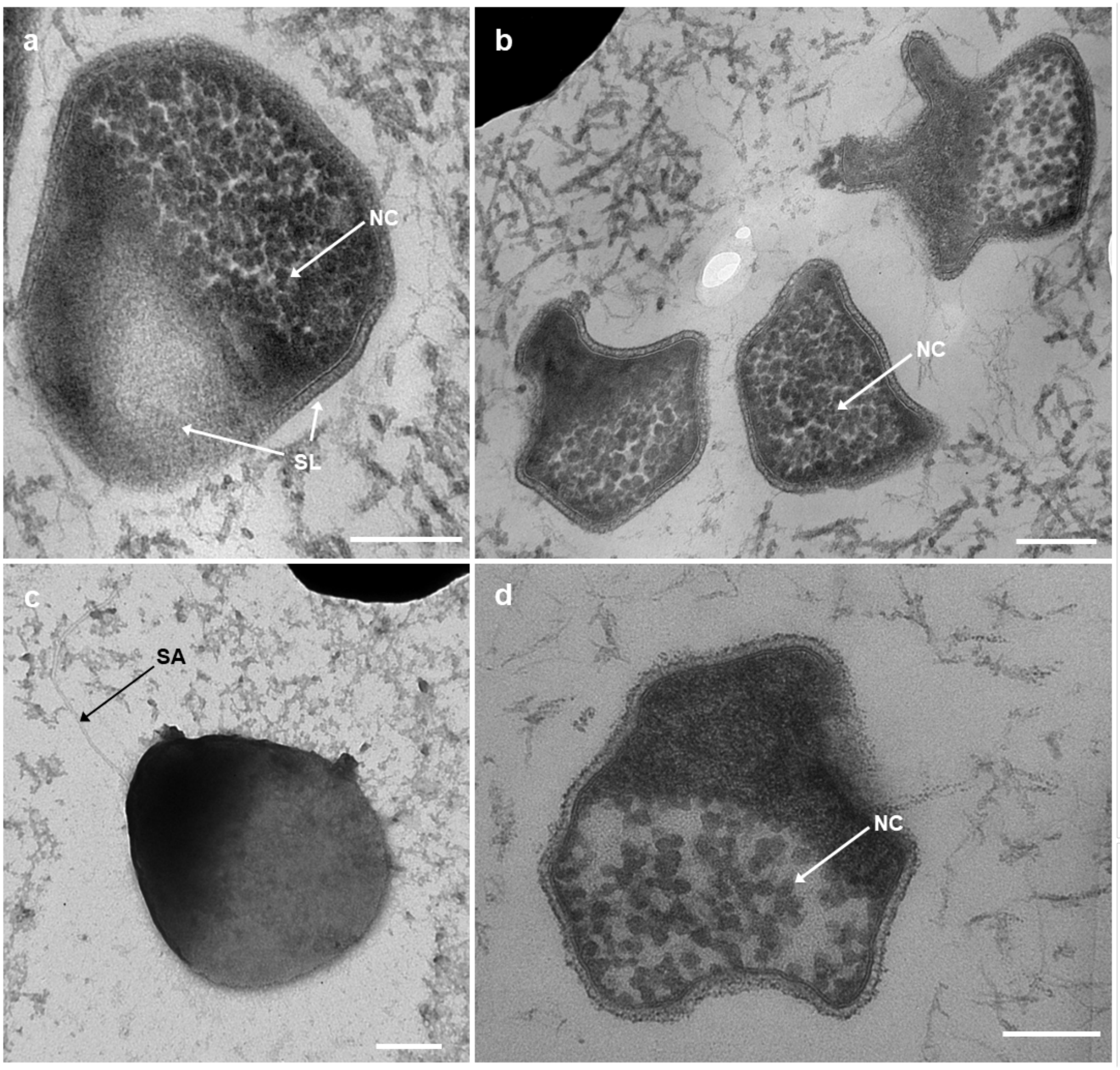

FIG S2 Transmission electron microscopy of MP-3918 (**a, b**) and AK-3817 (**c, d**) cells. S-layer (SL), nanocompartments (NC), surface appendage (SA).

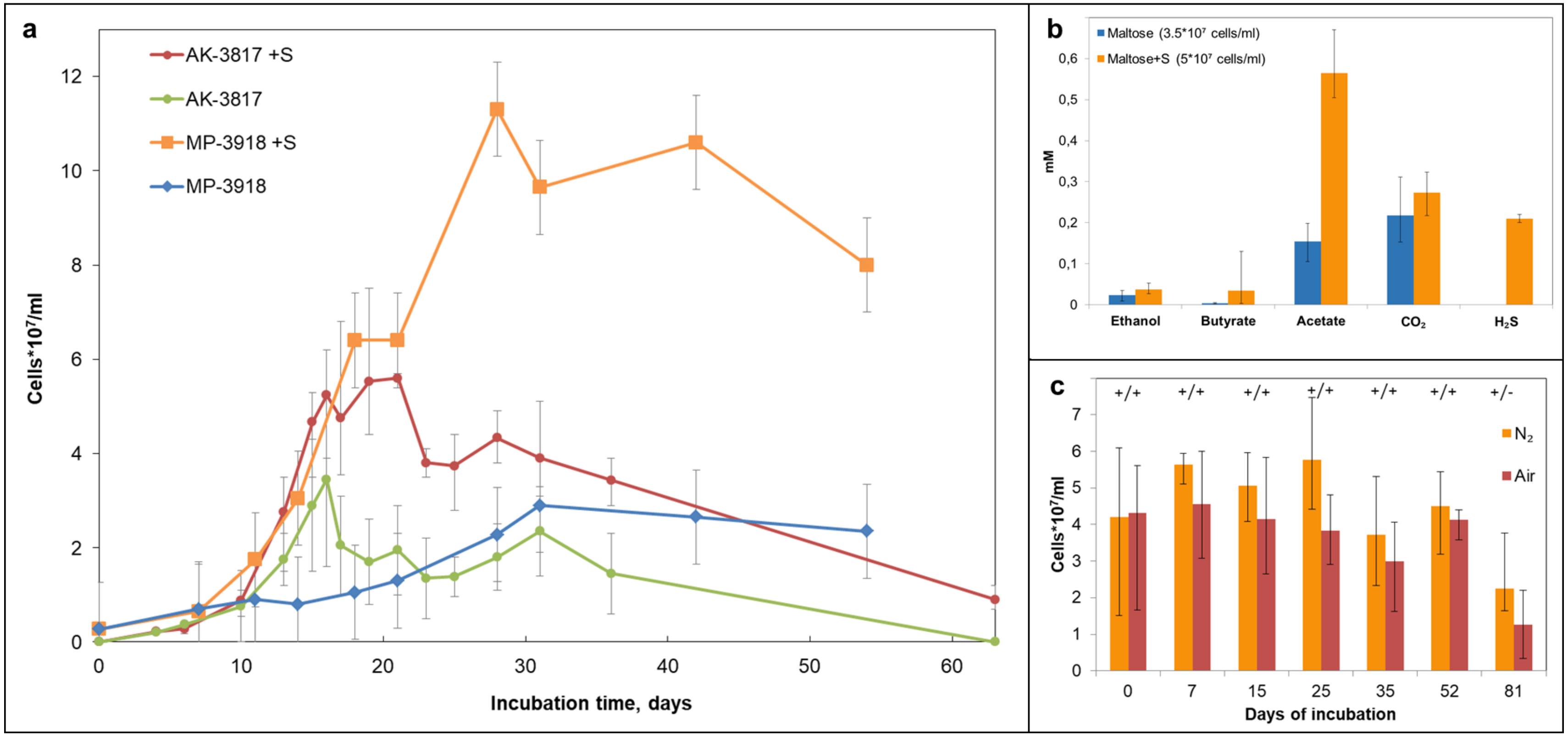

FIG S3 (a) Growth curves of *Tardisphaera* strains under optimal conditions (55°C/pH 4.0/starch for MP-3918 and 65°C/pH 4.0/galactomannan for AK-3817) with or without sulfur (each curve represents the average of three independent experiments). **(b)** The formation of products during the growth of the strain MP-3918 on maltose with or without sulfur (three independent replicates). **(c)** Oxygen tolerance experiment with the strain MP-3918. Cell yield during the incubation on medium, supplemented with Na_2S, elemental sulfur and starch, with (Air) or without O_2 access (N_2) to the gas phase (six independent experiments). The culture for the experiments was grown in advance during 21 days (the exponent stage) at strictly anaerobic conditions. +/+ means the culture has grown after the transferring to a fresh anaerobic medium in both experiment ("N_2" and "Air"); +/- means the culture has grown after the transferring to a fresh anaerobic medium only in "N_2" experiment. Error bars represent the range from the mean to the maximum or minimum value among replicates.

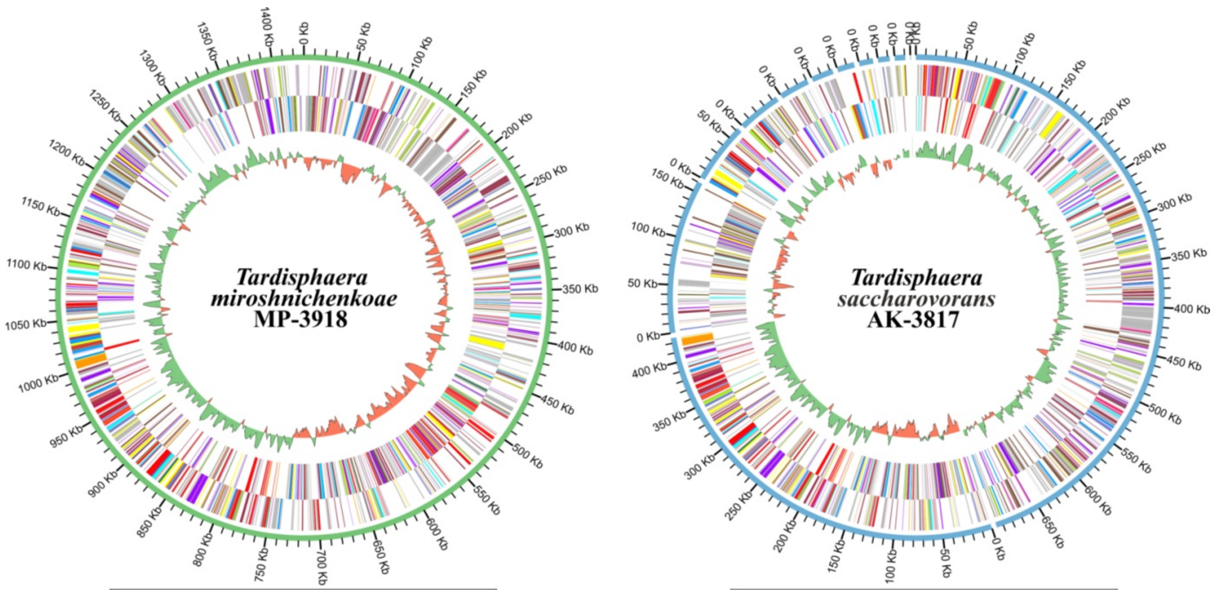

Genome size	1,429,880 bp
GC %	53.6%
Protein-coding genes	1,361
5S/16S/23S rRNA	1/1/1
tRNA	46
ncRNA	2
Pseudogenes	24

Genome size	1,498,369
GC %	51.9%
Protein-coding genes	1,382
5S/16S/23S rRNA	1/1/1
tRNA	46
ncRNA	2
Pseudogenes	29

- J Translation, ribosomal structure and biogenesis
- A RNA processing and modification
- K Transcription
- L Replication, recombination and repair
- B Chromatin structure and dynamics
- D Cell cycle control, cell division, chromosome partitioning
- Y Nuclear structure
- V Defense mechanisms
- T Signal transduction mechanisms
- M Cell wall/membrane/envelope biogenesis
- N Cell motility
- Z Cytoskeleton
- W Extracellular structures
- U Intracellular trafficking, secretion, and vesicular transport
- O Posttranslational modification, protein turnover, chaperones
- C Energy production and conversion
- G Carbohydrate transport and metabolism
- E Amino acid transport and metabolism
- F Nucleotide transport and metabolism
- H Coenzyme transport and metabolism
- I Lipid transport and metabolism
- P Inorganic ion transport and metabolism
- Q Secondary metabolites biosynthesis,transport,catabolism
- R General function prediction only
- S Function unknown

FIG S4 Chromosome maps and general genome characteristics of strains MP-3918 (left) and AK-3817 (right). The outer ring represents the length scale in Kb. There are two rings devoted to the functions of the annotated genes, the first ring comprises genes transcribed clockwise, while the second ring comprises genes transcribed anti-clockwise. The color of the genes corresponds to their COG function category. The histogram of GC-skew values is located within the diagram: values greater than zero are shaded in green, while values less than zero are shaded in orange.

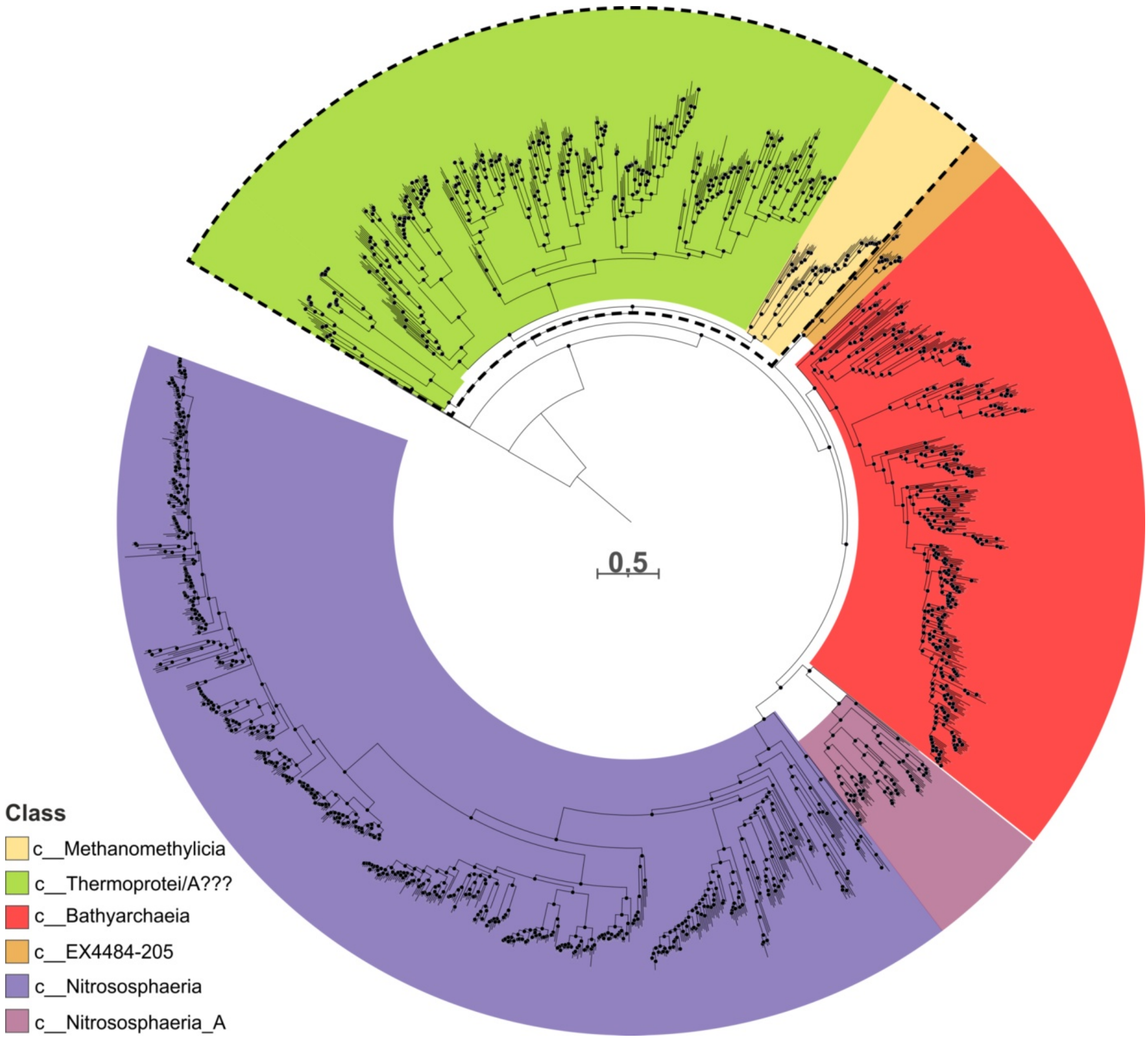

FIG S5 Phylogenomic analysis based on the "ar53" protein set showing diversity of the *Thermoproteota* phylum. Class assignment was taken according to the GTDB. The subtree bounded by a black dotted line is presented in Fig. 2a. The black circles at nodes indicate the percentage values of ultrafast bootstrap test (from 1000 replicates) are higher than 90%. *Halobacterium salinarum* DSM 3754 (GCA_008124605.1) and *Thermoplasma volcanium* GSS1 (GCA_000011185.1) were used as outgroups.

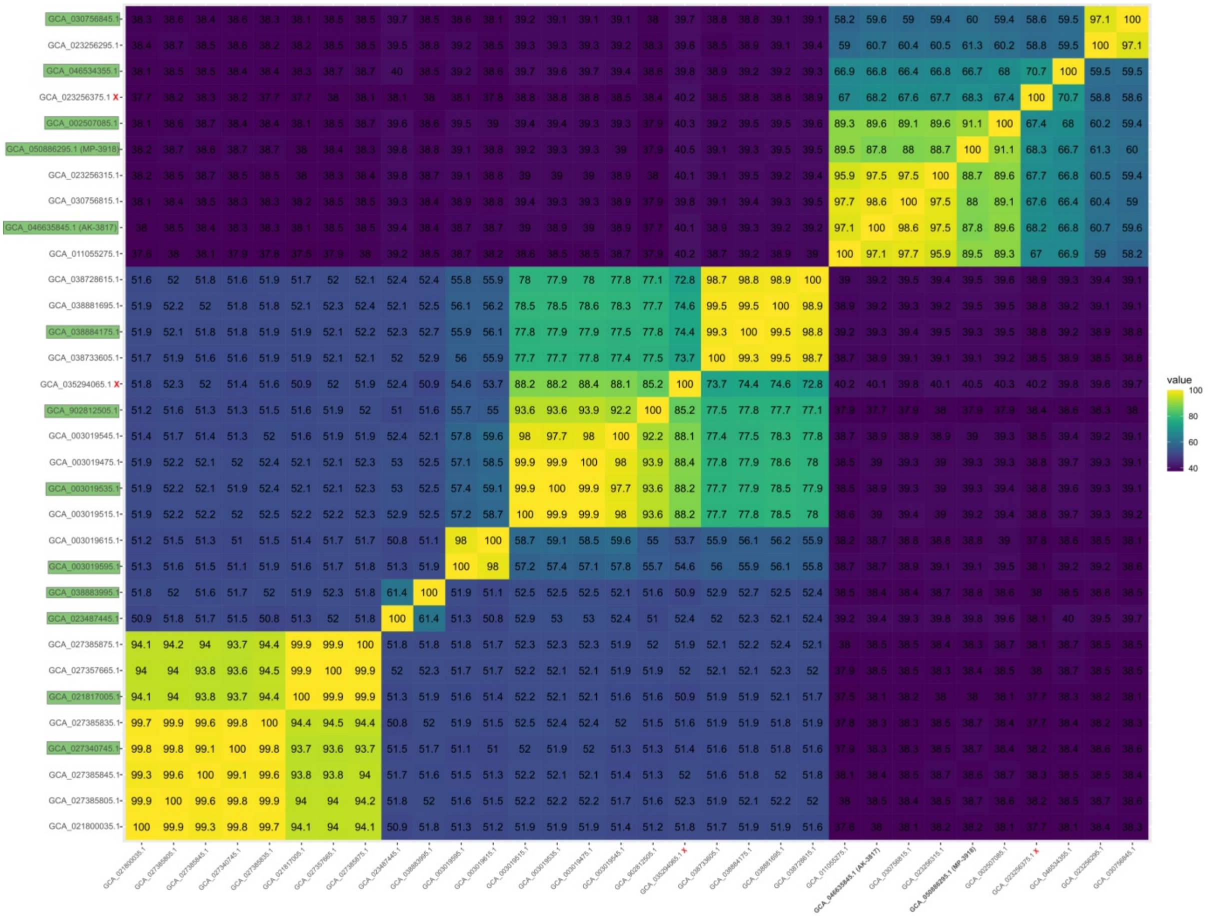

FIG S6 Matrix of average amino acid identities between representatives of the order *Tardispaherales*. Genomes inside the green boxes were taken as representatives for the species and were used for Figures 4 and 5, while genome marked by the red "X" were excluded from the genome analysis due to the low completeness.

a. Kamchatka

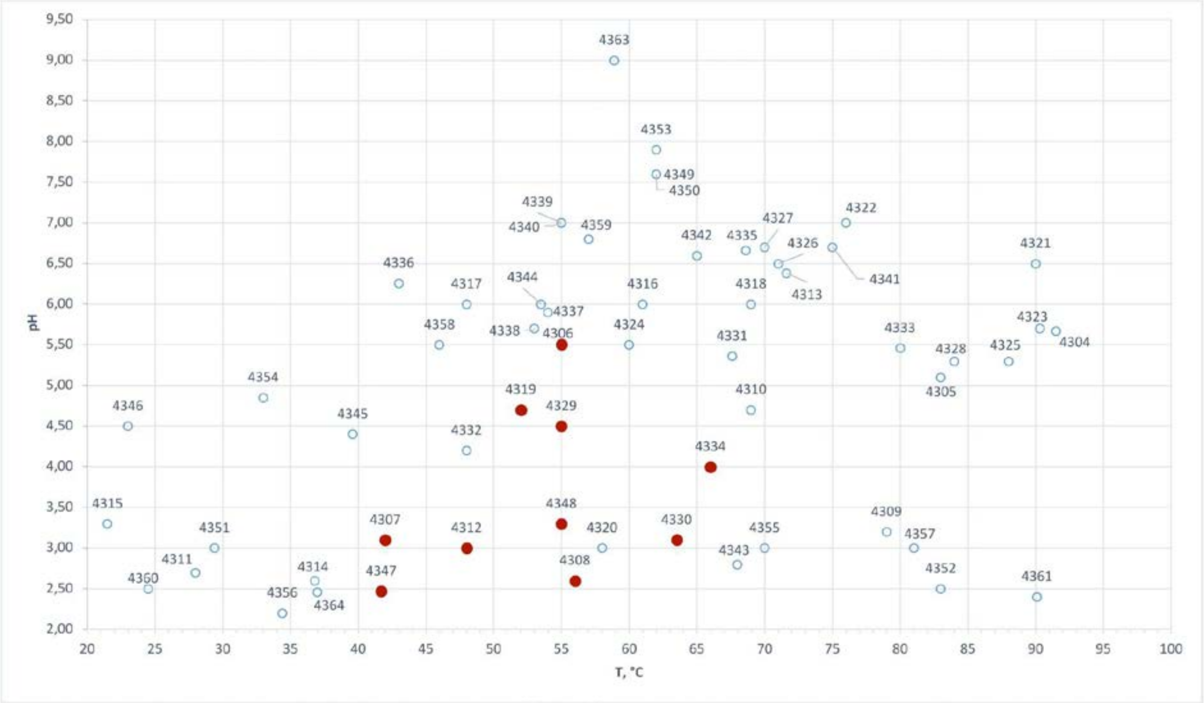

b. Kuril Islands

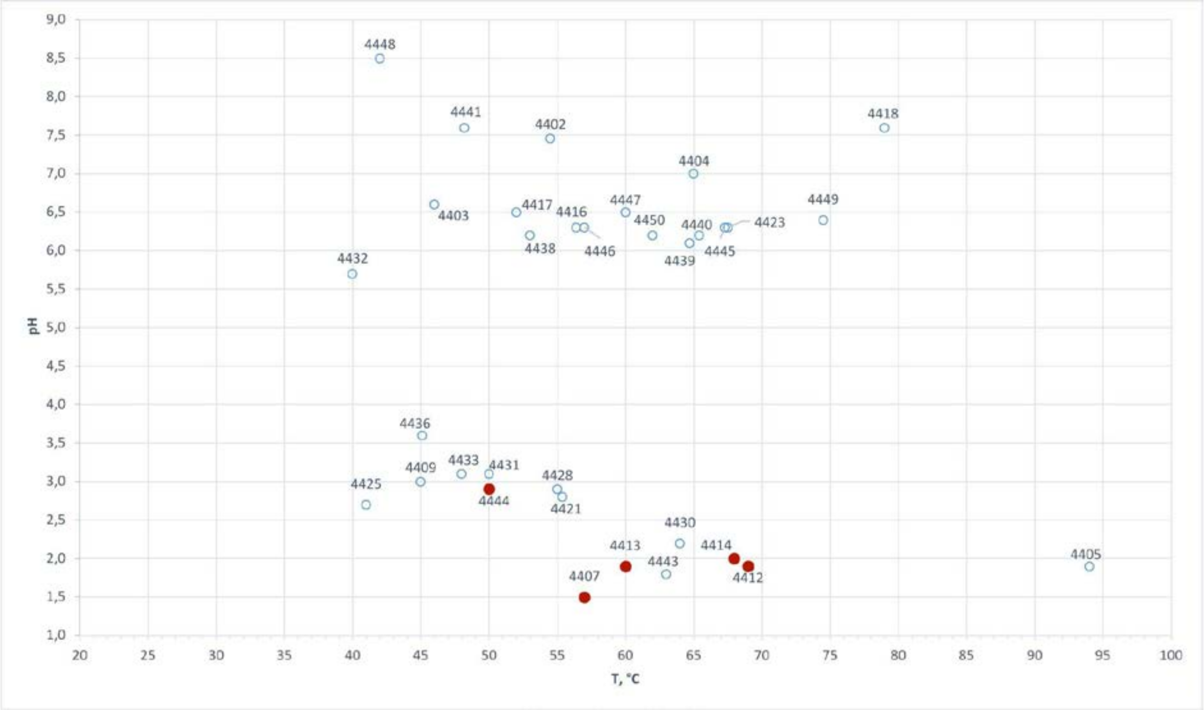

FIG S7 *Tardisphaerales* distribution (red dots) in thermal springs of Kamchatka **(a)** and Kuril Islands **(b).** Springs are labeled with ordinal numbers in the diagrams and are arranged according to the parameters of their temperature and pH.

Table S1. Intact polar lipid composition of strains MP-3918 and AK-3817

Polar headgroup 1	Core lipid	Polar headgroup 2	Observed accurate mass (*m/z*)	AEC ($[M+NH_4]^+$)	Δ mmu	Relative abundance (%)	
						MP3918	AK3817
None	Archaeol	n/a	670.7081	$C_{43}H_{92}O_3N$	0.4	2.4	15.3
Hexose	Archaeol	n/a	832.7604	$C_{49}H_{102}O_8N$	0.1	14.6	25.6
Di hexose	Archaeol	n/a	994.8125	$C_{55}H_{112}O_{13}N$	0.1	2.5	2.4
Di hexose	GDGT-0	Phosphoinositol	1885.473	$C_{104}H_{207}O_{24}NP$	1.3	11.9	12.3
	GDGT-1		1883.458	$C_{104}H_{205}O_{24}NP$	0.0	9.9	8.7
	GDGT-2		1881.444	$C_{104}H_{203}O_{24}NP$	-1.4	9.8	5.0
	GDGT-3		1879.429	$C_{104}H_{201}O_{24}NP$	-2.1	9.3	2.3
	GDGT-4		1877.414	$C_{104}H_{199}O_{24}NP$	-2.1	6.3	0.8
Tetra hexose	GDGT-0	None	1967.564	$C_{110}H_{216}O_{26}N$	-3.0	0.2	0.1
	GDGT-1		1965.545	$C_{110}H_{214}O_{26}N$	-0.1	0.4	0.1
	GDGT-2		1963.530	$C_{110}H_{212}O_{26}N$	-1.0	0.7	0.1
	GDGT-3		1961.514	$C_{110}H_{210}O_{26}N$	-0.2	1.6	0.1
	GDGT-4		1959.499	$C_{110}H_{208}O_{26}N$	-0.6	1.3	0.0
Di hexose	GDGT-0	None	1643.455	$C_{98}H_{196}O_{16}N$	-0.4	0.3	1.6
	GDGT-1		1641.439	$C_{98}H_{194}O_{16}N$	-0.1	1.0	3.9
	GDGT-2		1639.424	$C_{98}H_{192}O_{16}N$	-0.8	1.6	5.4
	GDGT-3		1637.406	$C_{98}H_{190}O_{16}N$	1.8	5.8	9.7
	GDGT-4		1635.390	$C_{98}H_{188}O_{16}N$	1.9	18.6	5.5

AEC = Assigned elemental composition; mmu = milli mass unit; Δ mmu = (measured mass – calculated mass) x 1000. n/a = not applicable.

Table S2. Phenotypic characteristics of strains MP-3918 and AK-3817

	MP-3918	AK-3817
Origin	solfatara soil, Kuril Islands, Russia	hot spring, Kamchatka, Russia
Morphology	irregular cocci 0.4-1.0 µm	irregular cocci 0.4-1.0 µm
min/opt/max T, °C	47/55-60/70	37/65/75
min/opt/max pH	3.0/3.9/4.7	3.0/3.9-4.0/4.7
O_2-tolerance	weak growth on Na_2S-free medium; tolerant to oxygen presence during 52-days incubation period	weak growth on Na_2S-free medium; tolerant to oxygen presence during 7-days incubation period
Autotrophy	-	-
Sulfur dependence	-	-
Substrates:		
Sugars		
cellobiose	-	+
fructose	+	+
maltose	+	+
galactose	+	+
glucose	-	+
lactose	+	+

xylose	+	+
Polysaccharides		
Cellulose (carboxymethylcellulose, Avicel, amorphous cellulose)	-	-
beta-glucan	+	+
dextrin	+	+
galactan	+	+
galactomannan	+	+
glucomannan	+	+
laminarin	+	+
mannan	+	+
pullulan	+	+
starch	+	+
xanthan gum	+	+
xylan	+	+
chitin	-	-
Proteinaceous substances		
gelatin	-	-
tryptone	-	-
yeast extract	-	+
Alcohols		
ethanol	-	-
mannitol	+	+
sorbitol	+	+
Organic acids		
acetate (with acceptor)	-	-
pyruvate (with or without acceptor)	-	-

Table S3. General characteristics of genome assemblies for strains MP-3918 and AK-3817

Characteristic	MP-3918	AK-3817
Size, bp	1,429,880	1,498,369
GC, %	53.6	51.9
Scaffold number	1	12
N50, bp	1,429,880	425,441
Completeness, %	92.13	91.36
Contamination, %	0.93	0.93
Protein-coding genes	1,361	1,382
Pseudogenes	24	29
Coding density, %	88.51	86.13
rRNA operon	1	1
tRNA genes	46	46
ncRNA genes	2	2

Table S4. Median values for relative evolutionary divergences (REDs) for different taxa levels estimated during our analysis

Taxon level	RED value
Phylum	0.227
Class	0.391
Order	0.533
Family	0.724
Genus	0.906

Table S5. Genetic determinants of distinct metabolic features of strains MP-3918 and AK-3817 (separate file **First cultivated Marsarchaeota_Table S5_Genetic determinants.xlsx**)

Table S6. CAZymes (excluding glycosyltransferases) encoded in genomes of *Tardisphaerales* representatives (separate file **First cultivated Marsarchaeota_Table S6_CAZymes.xlsx**)

Table S7. Possible horizontal gene transfers to *Tardisphaera* species based on the HGTector 2 results (separate file **First cultivated Marsarchaeota_Table S7_HGT.xlsx**)

Table S8. Metabolic potential comparison among *Tardisphaerales* representatives (separate file **First cultivated Marsarchaeota_Table S8_order_comparison.xlsx**)

Table S9. Global distribution of *Tardisphaerales* in acidic environments based on 16S rRNA gene amplicon, clone library and MAGs data sets (separate file **First cultivated Marsarchaeota_Table S9_Map.xlsx**)

Table S10. qPCR quantification of 16S rRNA gene copies of total bacteria and *Tardisphaerales* in wet sediments from thermal springs of Kamchatka and Kuril Islands

Region	Thermal Spring	Total number of 16S rRNA gene copies per 1 mL of wet sediment, $*10^5$	±SD	16S rRNA gene copies of *Tardisphaerales* per 1 mL of wet sediment, $*10^5$	±SD
Kamchatka	4306	17332.8	1387.9	132.5	8.2
Kamchatka	4307	6347.4	529.9	635.4	73.4
Kamchatka	4308	2376.9	219.3	82.6	38.5
Kamchatka	4312	155.2	4.7	35.4	12.8
Kamchatka	4319	122.7	32.6	1.7	0.0
Kamchatka	4329	4402.3	488.8	402.4	49.2
Kamchatka	4330	116.1	24.6	21.4	8.9
Kamchatka	4334	17.1	3.4	0.8	0.2
Kamchatka	4347	19233.7	5061.3	1654.6	619.5
Kamchatka	4348	78738.7	13620.2	1673.9	232.7
Kuril Islands	4407	1.4	0.3	0.1	0.0
Kuril Islands	4412	2.8	0.6	0.5	0.1

Kuril Islands	4413	61.6	3.4	25.1	7.7
Kuril Islands	4414	0.1	0.0	0.0	0.0
Kuril Islands	4444	1330.3	232.6	453.3	92.9

Table S11. Sugar metabolism pathways (EMP, ED and PPP) identified in genomes of cultivated thermoacidophilic and thermoacidotolerant prokaryotes (separate file **First cultivated Marsarchaeota_Table S11_Sugar_pathways.xlsx**)

Table S12. Characteristics of the sampling sites

MP-3918	AK-3817
Russian Federation, Kunashir (Kuril Islands), the Golovnin caldera, Cherepakhovoe field	Russian Federation, Kamchatka, the Uzon caldera, Orange field
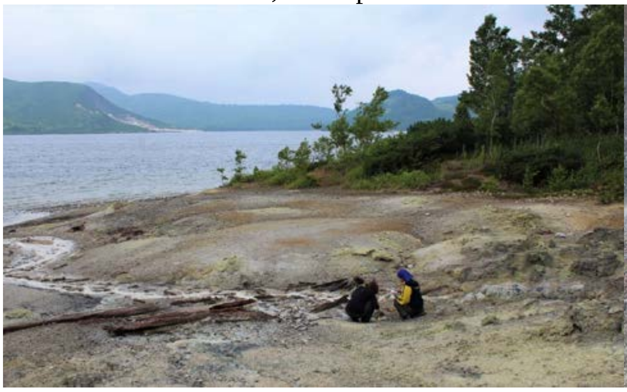	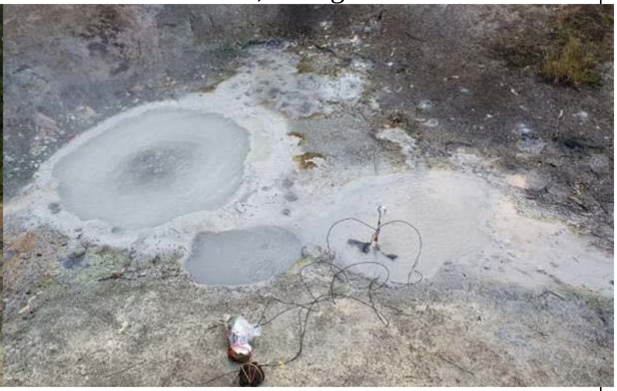
Small solfatara next to a stream, sampling is a grey sand near hot steam vent, yellow salt precipitation and algae encrustation observed around it<br>53°C, pH 3.0<br>N43°87’75.40’’<br>E145°51’32.86’’	A hot grey spring with active gas emission, turbid water and abundant sediments. The spring is located directly under a slope with high plants<br>53°C, pH 2.6, Eh +34 mV<br>N54°30’41.20’’<br>E160°00’43.00’’

Table S13. Primers used and designed in this work

Primer name	Sequence (5' > 3')	Reference
515F	GTGBCAGCMGCCGCGGTAA	[65]
Pro-mod-805R	GACTACNVGGGTMTCTAATCC	[66]
Tard-1138F	GGCAACGGCAGGTCAGCA	This work
Tard-1290R	TTCCAGGTTCACGAGGGC	This work

Table S14. Set of genome assemblies belonging to *Tardisphaerales* used for the analysis

Assembly	Completeness, %	Contamination, %
GCA_003019475.1	99.07	0
GCA_003019535.1	99.07	0
GCA_003019515.1	98.75	0
GCA_003019595.1	96.73	3.74
GCA_003019545.1	93.93	4.67
GCA_021800035.1	92.22	0.93
GCA_003019615.1	91.62	3.74
GCA_902812505.1	91.59	0.47
GCA_023487445.1	89.65	0.93
GCA_021817005.1	89.49	1.25
GCA_023256315.1	88.55	5.61
GCA_002507085.1	88.4	0
GCA_023256295.1	79.98	0
GCA_011055275.1	79.83	0.93
GCA_038881695.1	93.93	0.93
GCA_027385805.1	93.22	2.8
GCA_027385845.1	92.76	1.94
GCA_038728615.1	92.52	0
GCA_027340745.1	92.29	1.87
GCA_027357665.1	92.29	2.8
GCA_027385875.1	90.42	2.8
GCA_030756815.1	87.62	0.93
GCA_038733605.1	87.38	0.31
GCA_030756845.1	86.99	0
GCA_038884175.1	86.45	0.31
GCA_027385835.1	83.41	0.93
GCA_038883995.1	81.78	0.93
GCA_046534355.1	88.24	0.93

Supplementary File 1. Phylogenomic tree based on full “ar53”-based alignment of representative genomes available in GTDB r.226 (https://data.gtdb.ecogenomic.org/releases/release226/226.0/genomic_files_reps) constructed using IQtree with LG+F+I+G4 model and 1000 ultrafast bootstrap replications. The tree was decorated with the relative evolutionary divergence values using Phylorank (see details in the METHODS). Find in the separate **Supplementary File 1_ar53_gtdb_r226_IQtree_Tardisphaeria.red_decorated.svg.**
