# Supplementary material for: Polysaccharide-degrading archaea dominate acidic hot springs: genomic and cultivation insights into a novel Thermoproteota lineage
Source: mSystems. 2025 Sep 22;10(10):e00710-25. doi: 10.1128/msystems.00710-25 (PMC12542729; doi:10.1128/msystems.00710-25)
Supplement: Collection Cetrificates — Collection certificates of strain MP-3918 and AK-3817 deposition. [file msystems.00710-25-s0003.pdf]

MINISTRY OF SCIENCE AND HIGHER EDUCATION
OF THE RUSSIAN FEDERATION
Federal Research Center
Pushchino Scientific Center for Biological Research of the Russian Academy of Sciences
(PSCBR RAS)
SKRYABIN INSTITUTE OF BIOCHEMISTRY AND PHYSIOLOGY OF MICROORGANISMS
ALL-RUSSIAN COLLECTION OF MICROORGANISMS

ВКМ
Всероссийская коллекция микроорганизмов
VKM

✉ pr. Nauki, Pushchino, Moscow region, Russia, 142290
☎ +7(4967) 73-09-24; Факс +7(495)956-33-70
E-mail: VKM@pbcras.ru

CERTIFICATE OF DEPOSITION AND AVAILABILITY OF A MICROORGANISM

This is to certify that the following microorganism has been deposited into the All-Russian Collection of Microorganisms (VKM) by Dr Maria Prokofeva (Winogradsky Institute of Microbiology «Federal Research Centre «Fundamentals of Biotechnology» of the Russian Academy of Sciences»):

Tardisphaera miroshnichenkoae **strain MP-3918^T under the number**

VKM B-3629^T

This strain was deposited in VKM on April 04, 2022

The strain is viable and will be available to the public after publication by the author(s) of the scientific paper that describes this taxon.

Dr Viktoria Shcherbakova,
Curator responsible for the strain
August 20, 2025

MINISTRY OF SCIENCE AND HIGHER EDUCATION
OF THE RUSSIAN FEDERATION
Federal Research Center
Pushchino Scientific Center for Biological Research of the Russian Academy of Sciences
(PSCBR RAS)
SKRYABIN INSTITUTE OF BIOCHEMISTRY AND PHYSIOLOGY OF MICROORGANISMS
ALL-RUSSIAN COLLECTION OF MICROORGANISMS

✉ pr. Nauki, Pushchino, Moscow region, Russia, 142290
☎ +7(4967) 73-09-24; Факс +7(495)956-33-70
E-mail: VKM@pbcras.ru

CERTIFICATE OF DEPOSITION AND AVAILABILITY OF A MICROORGANISM

This is to certify that the following microorganism has been deposited into the All-Russian Collection of Microorganisms (VKM) by Alina Karaseva (Winogradsky Institute of Microbiology «Federal Research Centre «Fundamentals of Biotechnology» of the Russian Academy of Sciences»):

Tardisphaera saccharovorans **strain AK-3817^T under the number**

VKM B-3679^T

This strain was deposited in VKM on January 09, 2023

The strain is viable and will be available to the public after publication by the author(s) of the scientific paper that describes this taxon.

Dr Viktoria Shcherbakova,
Curator responsible for the strain
August 20, 2025

中国微生物菌种保藏管理委员会普通微生物中心
China General Microbiological Culture Collection Center (CGMCC)

Address: Institute of Microbiology, Chinese Academy of Sciences, Datun Road, Chaoyang District, Beijing 100101, China
Telephone: 86-10-64807355 Fax: 86-10-64807288 E-mail: cgmcc@im.ac.cn Homepage: www.cgmcc.net

保藏证明

CERTIFICATION OF DEPOSIT

CGMCC 1.18047

1. Name and address of the depositor or agent

Prokofeva M.I.
Winogradsky Institute of Microbiology, FRC Biotechnology RAS

2. Strain reference given by depositor

AK-3817

3. Deposited microorganisms appended

☐ Scientific description

■ Proposed taxonomic name

Tardisphaera saccharovorans

4. The deposited microorganism has been received and numbered as CGMCC 1.18047 in July, 2023. The strain has been checked for viability in the CGMCC and is stored using one of the standard methods used in the CGMCC.

5. This strain is available in the public accessible section of the CGMCC and restrictions have not been placed on access. It will be included in the published and online catalogue after publication of this number by the authors.

Signature of Director of CGMCC Ya-Jing YU

Date January 4, 2023

中国微生物菌种保藏管理委员会普通微生物中心
China General Microbiological Culture Collection Center (CGMCC)

Address: Institute of Microbiology, Chinese Academy of Sciences, Datun Road, Chaoyang District, Beijing 100101, China
Telephone: 86-10-64807355 Fax: 86-10-64807288 E-mail: cgmcc@im.ac.cn Homepage: www.cgmcc.net

保藏证明

CERTIFICATION OF DEPOSIT

CGMCC 1.18048

1. Name and address of the depositor or agent

Prokofeva M.I.
Winogradsky Institute of Microbiology, FRC Biotechnology RAS

2. Strain reference given by depositor

MP-3918

3. Deposited microorganisms appended

☐ Scientific description

■ Proposed taxonomic name

Tardisphaera miroshnichenkoae

4. The deposited microorganism has been received and numbered as CGMCC 1.18048 in July, 2023. The strain has been checked for viability in the CGMCC and is stored using one of the standard methods used in the CGMCC.

5. This strain is available in the public accessible section of the CGMCC and restrictions have not been placed on access. It will be included in the published and online catalogue after publication of this number by the authors.

Signature of Director of CGMCC Ya-Jing YU

Date January 4, 2024
